# *Toxoplasma gondii* GRA7-Targeted ASC and PLD1 Promote Antibacterial Host Defense via PKCα

**DOI:** 10.1371/journal.ppat.1006126

**Published:** 2017-01-26

**Authors:** Hyun-Jung Koh, Ye-Ram Kim, Jae-Sung Kim, Jin-Seung Yun, Kiseok Jang, Chul-Su Yang

**Affiliations:** 1 Department of Molecular and Life Science, College of Science and Technology, Hanyang University, Ansan, S. Korea; 2 Department of Pathology, College of Medicine, Hanyang University, Seoul, S. Korea; Harvard School of Public Health, UNITED STATES

## Abstract

Tuberculosis is a global health problem and at least one-third of the world’s population is infected with *Mycobacterium tuberculosis* (MTB). MTB is a successful pathogen that enhances its own intracellular survival by inhibiting inflammation and arresting phago-lysosomal fusion. We previously demonstrated that *Toxoplasma gondii* (*T*. *gondii*) dense granule antigen (GRA) 7 interacts with TNF receptor-associated factor 6 via Myeloid differentiation primary response gene 88, enabling innate immune responses in macrophages. To extend these studies, we found that GRA7 interacts with host proteins involved in antimicrobial host defense mechanisms as a therapeutic strategy for tuberculosis. Here, we show that protein kinase C (PKC)α-mediated phosphorylation of *T*. *gondii* GRA7-I (Ser52) regulates the interaction of GRA7 with PYD domain of apoptosis-associated speck-like protein containing a carboxy-terminal CARD, which is capable of oligomerization and inflammasome activation can lead to antimicrobial defense against MTB. Furthermore, GRA7-III interacted with the PX domain of phospholipase D1, facilitating its enzyme activity, phago-lysosomal maturation, and subsequent antimicrobial activity in a GRA7-III (Ser135) phosphorylation-dependent manner via PKCα. Taken together, these results underscore a previously unrecognized role of GRA7 in modulating antimicrobial host defense mechanism during mycobacterial infection.

## Author Summary

We previously demonstrated that *Toxoplasma gondii* (*T*. *gondii*) dense granule antigen (GRA) 7 interacts with TRAF6 via MyD88, enabling innate immune responses in macrophages and effective protection against *T*. *gondii* infection *in vivo*. However, its exact role and how it regulates host innate immune responses have not been fully explained. Herein, we show that PKCα-mediated phosphorylation of GRA7 is essential for the interaction between GRA7 and ASC or PLD1, which can promote antimicrobial defense against *Mycobacterium tuberculosis* (MTB). Notably, PKCα specifically phosphorylated Ser52 and Ser135 of GRA7 *in vitro* and *in vivo*, indicating that GRA7 is a substrate of PKCα. The N-terminal of GRA7 (GRA7-I) was sufficient for interaction with the PYD domain of ASC, which is capable of ASC oligomerization and inflammasome activation. Furthermore, GRA7-III interacted with the PX domain of PLD1, facilitating its enzyme activity, phago-lysosomal maturation, and subsequent antimicrobial activity in a GRA7 phosphorylation-dependent manner. Interestingly, phosphomimetic mutation in GRA7 overcame the need for PKCα. Collectively, these results provide novel insight into how GRA7 can promote ASC and PLD1 activation in a PKCα-dependent manner as an antimicrobial host defense mechanism.

## Introduction

Tuberculosis (TB) is an infectious disease caused by *Mycobacterium tuberculosis* (MTB) [1]. The World Health Organization reported that in 2014, 9.6 million cases and 1.5 million deaths were globally [2]. Recent developments in TB drug-development strategies (including new and repurposed antimicrobials and host-directed drugs) have produced new regimens to shorten treatment duration, improve outcomes of TB treatment such as, prevent resistance, reduce lung injury by promoting autophagy, antimicrobial peptide production, and other macrophage effector mechanisms, as well as inhibiting mechanisms causing lung inflammation and matrix destruction [1,3–5]. A wide range of candidate host-directed therapies (HDTs)-including new and repurposed drugs, biologics, and cellular therapies-have been proposed to accelerate eradication of infection and overcome the problems associated with current treatment regimens.

Recent studies have revealed the intracellular signaling pathways that govern the outcome of the innate immune response to mycobacteria infection and antibacterial defense [6–11]. First, the NLRP3 inflammasome complex, an intracellular protein complex consisting of the sensor NACHT, LRR and PYD domains-containing protein 3 (NLRP3), the adaptor apoptosis-associated speck-like protein containing a carboxy-terminal CARD (ASC), and pro-caspase-1 regulates IL-1β and IL-18 processing [10–12]. Jayaraman *et al*. showed that IL-1β directly promotes antimicrobial immunity in murine and human macrophages by regulating TNFR signaling and caspase-3 activation against MTB infection [10]. Verway *et al*. showed that 1-25-Dihydroxyvitamin D (1,25D) enhances IL-1β signaling from MTB-infected macrophages, inducing antimicrobial peptide DEFB4/HBD2 in primary lung epithelial cells, which in turn helps control MTB [11]. Second, host phospholipids play a critical role in the activation of the antimicrobial innate immune response [13]. Phospholipase D (PLD), which has two isoforms (PLD1 and PLD2) catalyzes the hydrolysis of the membrane phospholipid, phosphatidylcholine, to generate the metabolically active phosphatidic acid (PA) [14]. PLD1 is activated by arf-, ral-, and rho-family GTPases, and protein kinase C (PKC) α, while PLD2 activity is elevated by fatty acids [15]. Interestingly, MTB, unlike the nonpathogenic *M*. *smegmatis*, inhibits PLD activation during phagocytosis, a process that is associated with intracellular survival of the pathogen [6]. Garg *et al*. showed that Natural lysophospholipids promote MTB-induced *in vitro* PLD-dependent phagolysosome maturation and PLD-dependent intracellular killing of MTB in human macrophages [8] and the type II alveolar epithelial cell line A549 [9]. Third, recent studies have highlighted the role of protein kinases in the biology and pathogenesis of mycobacteria. The members of the PKC-family of proteins are classified into three groups, based on the mechanisms regulating their activation in response to different stimuli [7,16]. Holm *et al*. showed that PKCα regulates phagocytosis and the biogenesis of phagolysosomes by promoting the interaction of phagosomes with late endosomes and lysosomes [16]. Furthermore, PKCα also plays an important role in the killing of MTB in human macrophages [7]. Collectively, these infection-induced signaling pathways suggest possibilities for the development of novel therapeutic modalities for tuberculosis that target the intracellular signaling pathways permitting the replication of this nefarious pathogen. However, the roles of MTB-infection signal-dependent HDTs involved in host innate immune responses and their regulatory mechanisms have not yet been fully elucidated.

In a previous study, we demonstrated that *T*. *gondii* GRA7/MyD88-dependent NF-κB activation is essential for the activation of TNF receptor-associated factor 6 (TRAF6) and ROS generation, and enhances the release of inflammatory mediators. We also found that GRA7 stimulation led to physical and functional associations between GRA7 and TRAF6, resulting in crucial protective efficacy against *T*. *gondii* infection *in vivo* [17]. It remains to be seen whether GRA7 targeting can be used as a therapeutic strategy for infectious diseases. In this study, we further investigated the intracellular regulatory network of *T*. *gondii* GRA7-induced ASC, PLD1, and PKCα signaling pathways to help identify novel therapeutic modalities for tuberculosis. We found that the PKCα-mediated phosphorylation of GRA7 was essential for interaction between GRA7 and ASC or PLD1, which contributes to antimicrobial defense against MTB *in vitro* and *in vivo*. Our findings demonstrate that GRA7-I and -III play fine-tuning roles in the activation of HDTs and innate immune machineries through direct binding with ASC or PLD1 and may provide a unique opportunity for urgently needed therapeutic interventions against tuberculosis.

## Materials and Methods

### Ethic statement

All animal experimental procedures were reviewed and approved by the Institutional Animal Care and Use Committee of Hanyang University (protocol 2014–0207) and Bioleaders Corporation (Daejeon, Korea, protocol BLS-ABSL3-13-11). All animal experiments were performed in accordance with Korean Food and Drug Administration (KFDA) guidelines.

### *M*. *tuberculosis* culture

Cultures of MTB H37Rv (provided by Dr. R. L. Friedman, University of Arizona, Tucson, AZ) were prepared as described previously [1]. The effective concentration of lipopolysaccharide was <50 pg/ml in those experiments, with a bacterium-to-cell ratio of 10:1. For all assays, mid-log phase bacteria (absorbance 0.4) were used. Bacterial strains were divided into 1-ml aliquots and stored at -70°C.

### Mice and cells

Wild-type C57BL/6 mice were purchased from Orient Bio (Gyeonggi-do, Korea). PKCα^-/-^ (B6;129-Prkca^tm1Jmk^/J, 009068) and PLD1^-/-^ (B6.Cg-Pld1^tm1.1Gbp^/J, 028665) mice were obtained from Jackson Laboratory. All animals were maintained in a specific pathogen-free environment. HEK293T cells (ATCC-11268; American Type Culture Collection) were maintained in DMEM (Invitrogen) containing 10% FBS (Invitrogen), sodium pyruvate, nonessential amino acids, penicillin G (100 IU/ml), and streptomycin (100 μg/ml). Human monocytic THP-1 (ATCC TIB-202) cells were grown in RPMI 1640/glutamax supplemented with 10% FBS and treated with 20nM PMA (Sigma-Aldrich) for 24 h to induce their differentiation into macrophage-like cells, followed by washing three times with PBS. Primary bone marrow–derived macrophages (BMDMs) were isolated from C57BL/6 mice and cultured in DMEM for 3–5 d in the presence of M-CSF (R&D Systems, 416-ML), as described previously [12].

### *M. tuberculosis* infection *in vitro* and *in vivo*

For *in vitro* experiments, cells were infected with MTB for 2–4 h. Then, cells were washed with PBS to remove extracellular bacteria, supplied with fresh medium, and incubated at 37°C for indicated time points. For *in vivo* experiments, C57BL/6 mice were i.v. injected with MTB (1×10^6^ CFU/mouse). After 3 wks of infection, mice injected intraperitoneally with rGRA7 proteins for 7 consecutive days. After 1 wk of treatment, mice were sacrificed for harvesting of the lungs, spleens, and livers. Mice were maintained in biosafety level 3 laboratory facilities.

### Reagents, plasmids, and abs

CIP (P4978) and DMSO were purchased from Sigma-Aldrich. PKCα (C2-4) inhibitor peptide (17478) was purchased from Cayman Chemical. Flag-PKCα, -β, -δ, and -ξ plasmids were a generous gift from Dr. D. Zhou (Xiamen University, China). The GST-tagged GRA7 and truncated mutant genes were described previously [17]. V5-tagged AC or AU1-PLD1 and truncated mutant genes were cloned into the XbaI and BamHI sites in pcDNA3.0. All constructs were sequenced using an ABI PRISM 377 automatic DNA sequencer to verify 100% correspondence with the original sequence. Specific antibodies against phospho-(Thr147)-PLD1 (3831), phospho-(Ser561)-PLD2 (3834), PLD1 (3832), PLD2 (13904), PKCα (2056), PKCγ (43806), and NLRP4 (12421) were purchased from Cell Signaling Technology. Antibodies specific for actin (I-19), ASC (N-15-R), IL-18 (H-173-Y), TRAF6 (H-274), caspase-1 p10 (M-20), Rab5 (D-11), Rab7 (H-50), LAMP1 (E-5), LAMP2 (H4B4), Tubulin (B-5-1-2), Calnexin (H-70), FACL4 (N-18), VDAC (B-6), His (His17), V5 (C-9), Flag (D-8), and GST (B-14) were purchased from Santa Cruz Biotechnology. AU1 (GTX23402) and PKCβI (A10-F) were purchased from GenenTex and Antibodies-online Inc., respectively. IL-1β (AF-401-NA) and NLRP3 (AG-20B-0014) were from R&D Systems and Adipogen, respectively.

### Immunoblot analysis and immunoprecipitation

THP-1, 293T, and BMDMs were treated as indicated and processed for analysis by Western blotting, co-immunoprecipitation, and GST pulldown as previously described [17,18].

### Confocal fluorescence microscopy

Immunofluorescence analysis was performed as described previously [1]. The cells were fixed on coverslips with 4% (w/v) paraformaldehyde in PBS and then permeabilized for 10 min using 0.25% (v/v) Triton X-100 in PBS at 25°C. PLD1 or His was detected using a 1/100 dilution of the primary Ab for 1 h at 25°C. After washing, the appropriate fluorescently labeled secondary Abs were incubated for 1 h at 25°C. Slides were examined using laser-scanning confocal microscopy (model LSM 800; Zeiss). For colocalization analysis, the co-distribution of the PLD1 and GRA7 were quantified and validated statistically by Pearson coefficient, as specified by the ZEN 2009 software (version 5.5 SP1; Zeiss).

### Peptide spot arrays

Peptide arrays were synthesized using the SPOTs synthesis method and spotted onto a derivatized cellulose membrane (Intavis) in the presence of [γ-^32^P]ATP and calcium as described previously [19,20]. Peptide spot phosphorylation was quantified using phosphoimaging.

### Histology

For immunohistochemistry of tissue sections, mouse lungs were fixed in 10% formalin and embedded in paraffin. Paraffin sections (4 μm) were cut and stained with hematoxylin and eosin (H&E) [21].

### *In vitro* PLD activity assay

PLD activity was measured using the Amplex Red PLD assay kit (Molecular Probes, A12219) according to the manufacturer’s protocol. The resulting fluorescence was detected using a fluorescence microplate reader at an excitation of 530 nm and an emission of 590 nm.

### Recombinant GRA7 protein

The recombinant GRA7 protein was described previously [17]. GRA7s from amino acid residues 26–80, 26-80^S52A^, 26-80^S52D^, 120-150^S135A^ and 120-150^S135D^ were cloned with an N-terminal 6xHis-tag into the pRSFDuet-1 Vector (Novagen) and induced, harvested, and purified from *E*. *coli* expression strain BL-21 DE-3 pLysS, as described previously [17,22], following standard protocols recommended by Novagen.

### Supplemental Information

Supplemental experimental procedures and supplemental references.

### Statistical analysis

All data were analyzed by Student’s *t-*test with Bonferroni adjustment or ANOVA for multiple comparisons, and are presented as mean ± SD. Grubbs’ test was used for evaluating the outliers. Differences were considered significant at *p* <0.05.

## Results

### GRA7 associates with ASC and PLD1

**Fig 1:**
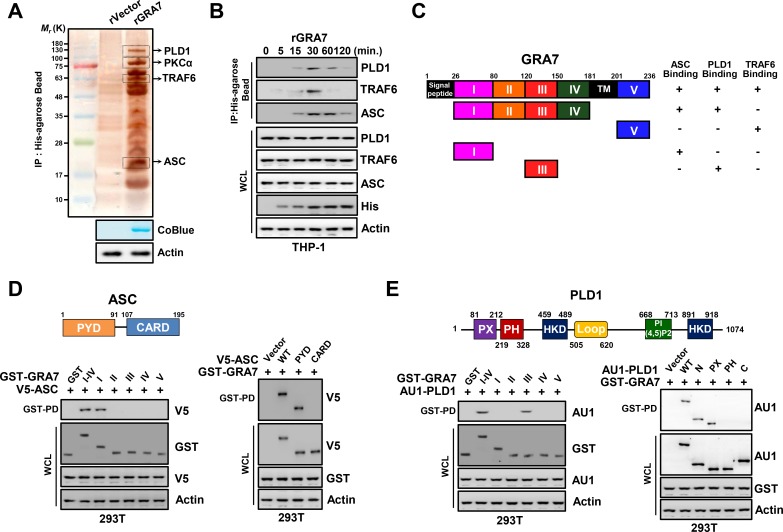
GRA7 interactions with ASC and PLD1. (**A**) Identification of PLD1, PKCα, TRAF6, and ASC by mass spectrometry analysis in THP-1 cell lysates incubated with a His-tagged rGRA7 (2 μg). CoBlue, staining of His-rGRA7 with Coomassie blue. Whole cell lysates (WCLs) were used for immuno blotting (IB) with αActin. (**B**) THP-1 cells were stimulated with rGRA7 (5 μg/ml) for the indicated times, followed by immunoprecipitation (IP) with αHis-agarose bead and IB with αPLD1, αTRAF6, αASC, αHis, and αActin. (**C**) Summary of the interactions of GRA7 WT and its mutants with ASC, PLD1, and TRAF6. The binding activities of GRA7 WT and mutants are summarized based on the results of **Fig 1D** and **1E**, **S2E Fig**, and [17]. (**D** and **E**) Binding mapping. Schematic diagram of the structures of ASC and PLD1 (upper). (**D**) At 48 hr post-transfection with mammalian GST or GST-GRA7 and truncated mutant constructs together with V5-ASC (left), V5, or V5-ASC constructs together with GST-GRA7 (right), 293T cells were used for GST pulldown, followed by IB with αV5. WCLs were used for IB with αGST, αV5 or αActin. (**E**) 293T cells were co-transfected with GST or GST-GRA7 and truncated mutant constructs together with AU1-PLD1 (left), or AU1 or AU1-PLD1 constructs together with GST-GRA7 (right), and subjected to GST pulldown, followed by IB with αAU1. WCLs were used for IB with αGST, αAU1 or αActin. The data are representative of four independent experiments with similar results (**A**, **B**, **D**, **E**).

To establish a role for GRA7 in intracellular signaling pathways as a therapeutic strategy for infectious diseases in macrophages, we investigated whether GRA7 interacts with molecules involved in innate immunity. GRA7 complexes were subjected to co-immunoprecipitation (co-IP) of recombinant GRA7 protein with THP-1 lysates. The purified GRA7 complexes retrieved several endogenous proteins selectively, as identified by mass spectrometry analysis, including PLD1 (124 K), PKCα (76 K), ASC (21 K), and TRAF6 (60 K) (Fig 1A and S1 Fig). Endogenous co-IP showed that GRA7 interacted strongly, although temporarily (from 15 to 60 min), with endogenous PLD1, TRAF6, and ASC but not with PLD2, NLRP3, or NLRC4 after stimulation with rGRA7 in THP-1 cells, and vice versa (Fig 1B, S2A and S2B Fig). As previously reported [17], GRA7 associated with TRAF6, based on their molecular weights and co-IP (Fig 1A–1C, S1 and S2A Figs).

To determine the mechanism by which rGRA7 interact with the intracellular protein, THP-1 cells were preincubated with cytochalasin D, which inhibits actin polymerization. Pretreatment with cytochalasin D completely blocked the phagocytic activities of rGRA7 and it binding with intracellular proteins (S2C and S2D Fig).

Structurally, GRA7 contains a signal sequence, N-terminal domains (I–IV), a transmembrane, and a C-terminal domain (V) (Fig 1C) [17]. In 293T cells, detailed mapping using various mammalian glutathionine S-transferase (GST)-GRA7 fusions and truncated mutants of V5-ASC indicated that the N-terminal I-domain (aa26-80) of GRA7 exhibited only minimal binding affinity to ASC and ASC carrying the N-terminal PYD domain (aa1-91) bound GRA7 as strongly as ASC WT (Fig 1C and 1D). GST pull-down assays using truncated mutants of GST-GRA7 mammalian fusions and AU1-PLD1 showed that the N-terminal III-domain (aa120-150) of GRA7 is required for its interaction with PX (aa81-212) of PLD1 (Fig 1C and 1E and S2E Fig), indicating that the interactions of GRA7 with ASC, PLD1, and TRAF6 are genetically separable (Fig 1). These results show that GRA7 interacts with ASC and PLD1 through its N-terminal I- and III-domains in macrophages, respectively.

### GRA7 interaction with ASC and PLD1 via PKCα

**Fig 2:**
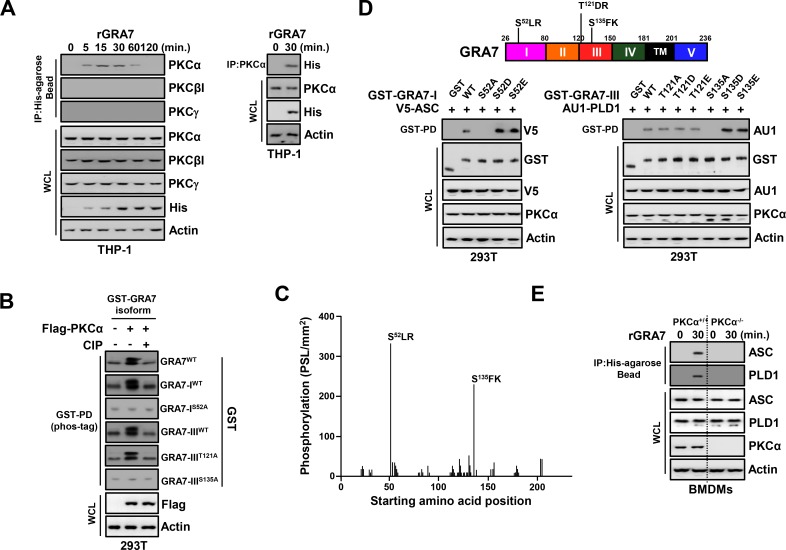
PKCα-dependent phosphorylation of GRA7 was essential for interaction with ASC and PLD1. (**A**) THP-1 cells were stimulated with rGRA7 (5 μg/ml) for the indicated times (left) or 30 min. (right), followed by IP with αHis-agarose bead (left) or αPKCα (right) and IB with αPKCα, αPKCβI, αPKCγ, αHis, and αActin. (**B**) Phos-tag and SDS-PAGE analyses of GST-GRA7, GRA7-I^WT^, GRA7-I^S52A^, GRA7-III^WT^, GRA7-III^T121A^, or GRA7-III^S135A^ expressed together with Flag-tagged PKCα in 293T cells left untreated (CIP-) or treated with calf intestinal alkaline phosphatase (CIP+), and subjected to GST pulldown, followed by IB with αGST. WCLs were used for IB with αFlag or αActin. (**C**) Mapping of PKCα phosphorylation sites on GRA7 by tiled peptide array analysis using purified recombinant PKCα. Phosphorylation intensity of 15-amino acid peptides that span full-length GRA7 and are each shifted by 3 amino acids was detected using MultiGauge version 3.0. The serines in the two peptides that showed a phosphorylation signal stronger than 100 PSL/mm^2^ are indicated above the corresponding peaks. (**D**) 293T cells were co-transfected with GST or GST-GRA7-I and truncated mutant constructs together with V5-ASC (left), or GST or GST-GRA7-III and truncated mutant constructs together with AU1-PLD1 (right), and subjected to GST pulldown, followed by IB with αV5 or αAU1. WCLs were used for IB with αGST, αV5, αAU1, αPKCα, or αActin. (**E**) BMDMs from PKCα^+/+^ and PKCα^-/-^ were stimulated with rGRA7 for 30 min., followed by IP with αHis-agarose bead and IB with αASC, αPLD1, αPKCα, and αActin. The data are representative of four independent experiments with similar results (**A** to **E**).

In addition to ASC and PLD1 binding, GRA7 also interacted with PKCα. Endogenous co-IP revealed a robust interaction between GRA7 and PKCα, but not PKCβI or PKCγ, after stimulation with rGRA7 in THP-1 cells, and vice versa (Fig 2A).

A large-scale proteomics analysis of the human kinome [23] and computational sequence analysis [24] predicted five PKC phosphorylation residues (S^52^LR, T^121^DR, S^135^FK, T^204^TR, S^209^PR) within the GRA7 N-terminal I, III domains, and C-terminal V-domain. To confirm that GRA7 was phosphorylated by PKCα, we used several strategies. First, we performed Phos-tag gel electrophoresis, which involves the use of a Phos-tag biomolecule that specifically binds phosphorylated proteins and retards their migration in the gel [25]. The results showed that GRA7 of wild-type, I-, and III-domains migrated more slowly and produced an ‘up-shifted’ band (as visualized by the Phos-tag labeling system for the analysis of phosphorylation, followed by SDS-PAGE) when co-expressed with PKCα, but when co-expressed with PKCβ, PKCδ, or PKCξ (Fig 2B and S3A Fig). Furthermore, we performed an *in vitro* phosphorylation assay using purified recombinant PKCα and a non-biased overlapping peptide array covering the entire GRA7 sequence [19,20]. From GRA7, two peptides (^49^PVD**S**LRPTNAGVDSK^73^ and ^121^TDRKVVPRKSEGKR**S**^135^) showed a phosphorylation signal >200 PSL/mm^2^ (Fig 2C). In contrast, none of the peptides spanning the C-terminal of GRA7 showed a significant phosphorylation signal. GRA7 has serine/threonine residues, and two peptides of GRA7 that were phosphorylated contained three potential phosphorylation sites (Fig 2C and 2D). Interestingly, the specific point mutation forms (I^S52A^ and III^S135A^) of GRA7 markedly decreased phosphorylation in Phos-tag gel electrophoresis, whereas the mutant III^T121A^ of GRA7 did not (Fig 2B). These results indicate that PKCα can specifically phosphorylate S52 and S135 residues of GRA7, demonstrating that GRA7 is a substrate of PKCα.

We next investigated whether phosphorylation of S52 and S135 of GRA7 was necessary for binding with ASC and PLD1, respectively. The point mutation (I^S52A^ and III^S135A^) of GRA7 markedly abolished its interaction with ASC and PLD1, suggesting that this interaction is S52- and S135-phosphorylation dependent (Fig 2D and S3B Fig). Furthermore, because phosphomimetic residues (aspartic acid or glutamic acid) do not fully approximate the electronegativity produced by phosphorylation, we employed the strategy of mutating amino acids to overcome the charge differential [20,26]. The GST pull-down assay showed that phosphomimetic mutants of GRA7 strongly bound to ASC and PLD1 compared to GRA7 WT, indicating that mimicking constitutively phosphorylated GRA7 overrode the need for PKCα function in the innate immune pathway. Consistent with the findings shown in Fig 2A–2D, GRA7 interaction with ASC and PLD1 was markedly decreased in BMDMs from PKCα^-/-^ mice, THP-1 from knock down with shRNA specific for PKCα (Fig 2E and S3C Fig), and BMDMs treated with a pharmacological inhibitor of PKCα upon rGRA7 stimulation (S3D Fig). Taken together, these data indicate that PKCα-mediated phosphorylation of GRA7 at Ser52 or Ser135 is essential for interactions between GRA7 and ASC or PLD1, respectively.

### GRA7-I induces activation of ASC-dependent inflammasome

**Fig 3:**
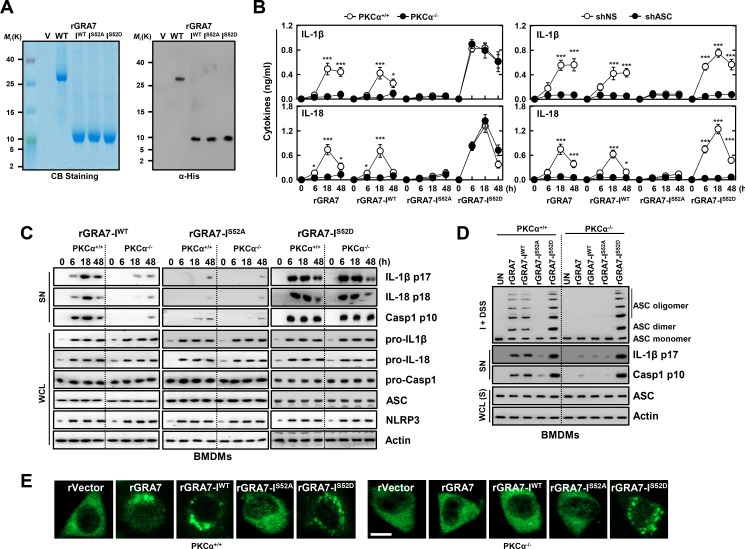
GRA7-I activated inflammasomes in an ASC-binding dependent manner. (**A**) Bacterially purified 6xHis-GRA7-I and its mutants were analyzed by Coomassie blue staining (left) or IB with αHis (right). (**B**) BMDMs from PKCα^+/+^ and PKCα^-/-^ (left) or BMDMs were transduced with lentivirus-shRNA-NS or lentivirus-shRNA-ASC (MOI = 100) with polybrene (8 μg/mL) (right) for 2 days, the cells were stimulated with rGRA7 (5 μg/ml) and its mutants for the indicated times and culture supernatants were harvested and analyzed for cytokine ELISA for IL-1β and IL-18. Data shown are the means ± SD of five experiments. Significant differences (*P < 0.05; ***P < 0.001) compared with PKCα^+/+^ or shRNA-NS. (**C**) IB analysis for IL-1β p17, IL-18 p18, or caspase-1 p10 in supernatants (SN), ASC, NLRP3, pro-IL-1β, pro-IL-18, or pro-caspase-1 in whole-cell lysates (WCL). Actin was used as a loading control. (**D**) IB analysis of lysates of BMDMs as in **B** and **C** solubilized with Triton X-100–containing buffer, followed by cross-linkage of insoluble fractions with disuccinimidyl suberate to capture ASC oligomers and analysis of those fractions (I + DSS) and soluble fractions (S) with antibody to ASC. Actin was used as a loading control. (**E**) Fluorescence confocal images showing formation of speck-like ASC pyroptosomes in BMDMs from PKCα^+/+^ and PKCα^-/-^ mice were stimulated with rGRA7 and its mutants for 18 h, fixed, immunostained with antibodies for ASC (Alexa 488). Scale bar, 10 μm. The data are representative of five independent experiments with similar results (**A** and **C**-**E**).

To examine the role of *T*. *gondii* GRA7-I in innate immune responses of macrophages, we generated bacterially purified His-tagged GRA7-I and its mutant proteins, as described previously [17,22]. The purified rGRA7-I (10 kDa) was confirmed through SDS-PAGE and immunoblotting analysis (Fig 3A). No significant difference compared to vector control observed for rGRA7-induced cytotoxicity in macrophages [17].

We showed previously that rGRA7-induced expression of pro-inflammatory cytokine genes and proteins including IL-1β, in macrophages [17] and NLRP3 inflammasomes involves a multimeric protein complex containing NLRP3 interacting the adaptor ASC and caspase-1 to induce the maturation of IL-1β and IL-18 [10,12]. To investigate the role of GRA7-I in the regulation of inflammasome activation, BMDMs from PKCα^+/+^ and PKCα^-/-^ mice were stimulated with rGRA7-I and its mutant proteins. In response to rGRA7-I and -WT, PKCα-deficient BMDMs showed significantly attenuated IL-1β and IL-18 production than WT BMDMs, but the phosphomimetic mutant (I^S52D^)-induced markedly increased secretion of IL-1β and IL-18 (Fig 3B and S4A Fig). Consistent with these results, the caspase-1 activation and IL-1β and IL-18 maturation observed in response to rGRA7-I and -WT proteins were significantly decreased in PKCα-deficient BMDMs, and the constitutively active form (I^S52D^) of GRA7 ‘rescued’ the PKCα deficiency (Fig 3C). Notably, the PKCα non-phosphorylatable mutant (I^S52A^) and shRNA-mediated reduction of endogenous ASC expression led to significant attenuation of IL-1β and IL-18 production (Fig 3B and S4B Fig) in an ASC-binding dependent manner.

Next, we determined whether ASC is substantially oligomerized and if the intracellular formation of ASC specks is dependent on GRA7-I interaction. In correlation with secretion of active caspase-1 and IL-1β, PKCα-deficient BMDMs showed markedly attenuated ASC oligomerization and speck formation compared to WT BMDMs, but the phosphomimetic mutant (I^S52D^) markedly increased both (Fig 3D and 3E and S4C Fig). Further, the intracellular interaction of GRA7 and ASC was confirmed by their co-localization after stimulation with rGRA7. Subcellular fractionation and co-IP analysis showed that GRA7-I associated with ASC and PKCα in the mitochondrial fraction in PKCα^+/+^ BMDMs. Notably, these binding patterns were increased by the phosphomimetic mutant (I^S52D^) in BMDMs from PKCα^+/+^ and PKCα^-/-^ mice (S4D Fig). These data suggest that GRA7-I acts as a positive regulator of ASC-dependent inflammasome activation via PKCα in mitochondria.

### GRA7-I-induced activation of ASC-dependent inflammasomes is important for resistance against MTB infection

**Fig 4:**
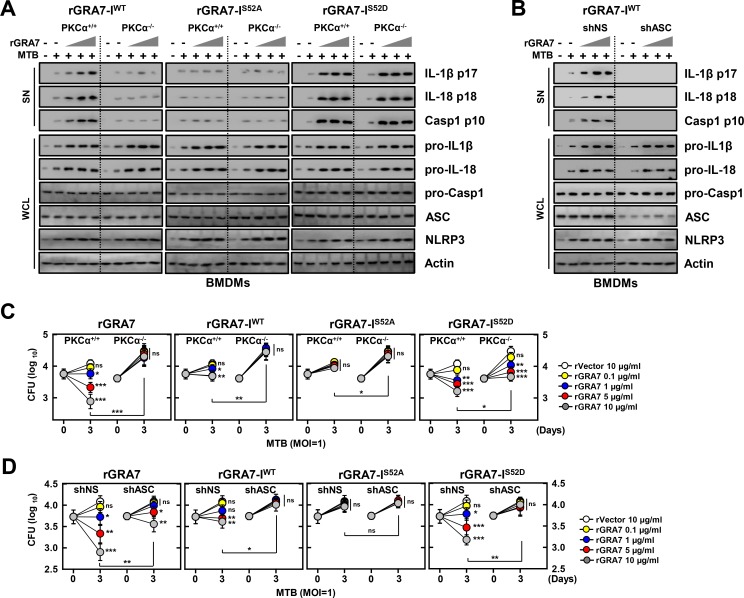
GRA7-I-induced inflammasome activation was required for antimicrobial activity in MTB-infected macrophages. (**A** and **B**) BMDMs from PKCα^+/+^ and PKCα^-/-^ mice (**A**) and BMDMs were transduced with lentivirus-shRNA-NS or lentivirus-shRNA-ASC for 2 days (**B**) infected with MTB (MOI = 1) for 4 h and then stimulated with rGRA7 (1, 5, 10 μg/ml) and its mutants for 18 h. IB analysis for IL-1β p17, IL-18 p18, or caspase-1 p10 in supernatants (SN), ASC, NLRP3, pro-IL-1β, pro-IL-18, or pro-caspase-1 in whole-cell lysates (WCL). Actin was used as a loading control. (**C** and **D**) Intracellular survival of MTB was assessed by CFU assay. BMDMs were infected with MTB for 4 h, followed by treatment with rGRA7, and then lysed to determine intracellular bacterial loads. The data are representative of five independent experiments with similar results (**A** and **B**). Data shown are the mean ± SD of five experiments (**C** and **D**). Significant differences (*P < 0.05; **P < 0.01; ***P < 0.001) compared with rVector. CFU, colony-forming units. ns, not significant.

IL-1β and IL-18 are cytokines that play crucial roles in host defense and inflammation [11,12]. We first measured caspase-1 activation and maturation of IL-1β and IL-18 induced by rGRA7 and its mutants in MTB-infected macrophages. rGRA7-I treatment increased inflammasome activity in MTB-infected macrophages in a dose, ASC-binding and PKCα phosphorylation-dependent manner. Importantly, treatment with the phosphomimetic mutant (I^S52D^) of GRA7 markedly amplified inflammasome activity in MTB-infected conditions in BMDMs from PKCα^+/+^ and PKCα^-/-^ mice (Fig 4A). The PKCα non-phosphorylatable mutant (I^S52A^) and the shRNA-mediated reduction of endogenous ASC expression led to significant attenuation of caspase-1 activation and maturation of IL-1β and IL-18 (Fig 4A and 4B) in an ASC-binding dependent manner.

IL-1β directly activates MTB–infected macrophages to restrict intracellular bacterial replication [10,27]. We examined whether rGRA7-induced antimicrobial activity was dependent on ASC-dependent inflammasome activation via PKCα in macrophages. The rGRA7-WT and -I-induced antimicrobial responses against MTB were significantly downregulated in BMDMs from PKCα^-/-^ mice and cells transduced with shASC in a dose-dependent manner (Fig 4C and 4D). Notably, the PKCα non-phosphorylatable mutant (I^S52A^) of GRA7 did not induced antimicrobial responses of MTB, compared with the WT- and rGRA7-I treatment, in BMDMs from PKCα^+/+^ mice. The phosphomimetic mutant (I^S52D^) of GRA7 markedly increased antimicrobial responses to MTB in dose-dependent manner, indicating that the constitutively active form (I^S52D^) of GRA7 partially ‘rescued’ the PKCα deficiency. No significant difference was observed for MTB growth in 7H9 broth with or without rGRA7 (S5 Fig), indicating that ASC-dependent inflammasome-derived IL-1β controls the outcome of MTB infection and is functionally linked via PKCα in macrophages.

### GRA7-III induces activation of PLD1

**Fig 5:**
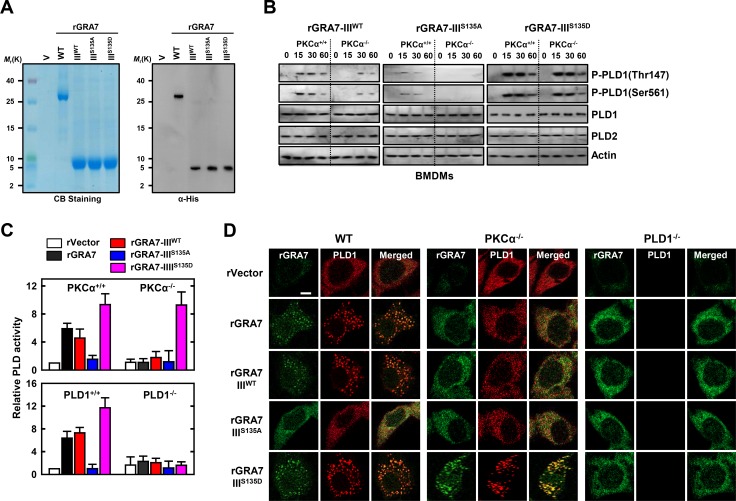
GRA7-III-induced activation of PLD1 was dependent on interaction with PLD1. (**A**) Bacterially purified 6xHis-GRA7-III and its mutants were analyzed by Coomassie blue staining (left) or IB with αHis (right). (**B**) BMDMs from PKCα^+/+^ and PKCα^-/-^ mice were stimulated with rGRA7-III (5 μg/ml) and its mutants for the indicated times, followed by IB to detect phosphorylated and total forms of PLD1 and PLD2. Actin was used as a loading control. (**C**) BMDMs were stimulated with rGRA7-III and its mutants for 30 min. and analyzed for PLD activity assay *in vitro*. Data shown are the means ± SD of five experiments. (**D**) Fluorescence confocal images in BMDMs were stimulated with rGRA7 and its mutants for 30 min., fixed, immunostained with antibodies for His (Alexa 488) and PLD1 (Alexa 568). Scale bar, 20 μm. The data are representative of three independent experiments with similar results (**A**, **B**, and **D**).

To examine the role of *T*. *gondii* GRA7-III in innate immune responses by macrophages, we generated bacterially purified His-tagged GRA7-III and its mutant proteins, as described previously [17,22]. The purified rGRA7-III (5 kDa) was confirmed through SDS-PAGE and immunoblotting analysis (Fig 5A).

As GRA7 associates with PLD1 but not PLD2 (Fig 1 and S1 Fig), we sought to determine whether PLD1 activation by GRA7-III was regulated by phosphorylation events in many cellular processes [15,28]. PLD1 activity is regulated by phosphorylation of Thr147 in the PX domain and Ser561 in the negative regulatory loop region of PLD1 by PKCα [15,28]. We first measured rGRA7-III-induced phosphorylation of PLD1 at Thr147 and Ser561 but not the PKCα non-phosphorylatable mutant (III^S135A^) of GRA7 in macrophages. Importantly, the phosphomimetic mutant (III^S135D^) of GRA7 treatment markedly amplified PLD1 activation in BMDMs from PKCα^+/+^ and PKCα^-/-^ mice (Fig 5B). Consistent with these results, PLD activity was significantly decreased by the PKCα non-phosphorylatable mutant (III^S135A^) and increased by the phosphomimetic mutant (III^S135D^) of GRA7 in BMDMs from PKCα^+/+^ and PKCα^-/-^ mice (Fig 5C). However, PLD activity was at the basal level in PLD1^-/-^ macrophages with the phosphomimetic mutant, indicating that phosphorylated GRA7-III interacted with activated PLD1 by PKCα and stimulated its enzymatic activity through the phosphorylation of PLD1 Thr147 and Ser561. Further, the intracellular interaction of GRA7 and PLD1 was confirmed by their co-localization after stimulation with rGRA7, as documented by immunostaining and image overlay (Fig 5D and S6 Fig). GRA7-III localized with PLD1 and PKCα in the cytoplasm, appearing as small speckles and punctate spots. Notably, these co-localization patterns were increased by the phosphomimetic mutant (I^S135D^) in BMDMs from PKCα^+/+^ and PKCα^-/-^ mice, but not PLD1^-/-^ mice. These data suggest that GRA7-III acts as a positive regulator of PLD1 activation via PKCα in macrophages.

### GRA7-III-induced phagosome maturation of PLD1 is important for resistance against MTB infection

**Fig 6:**
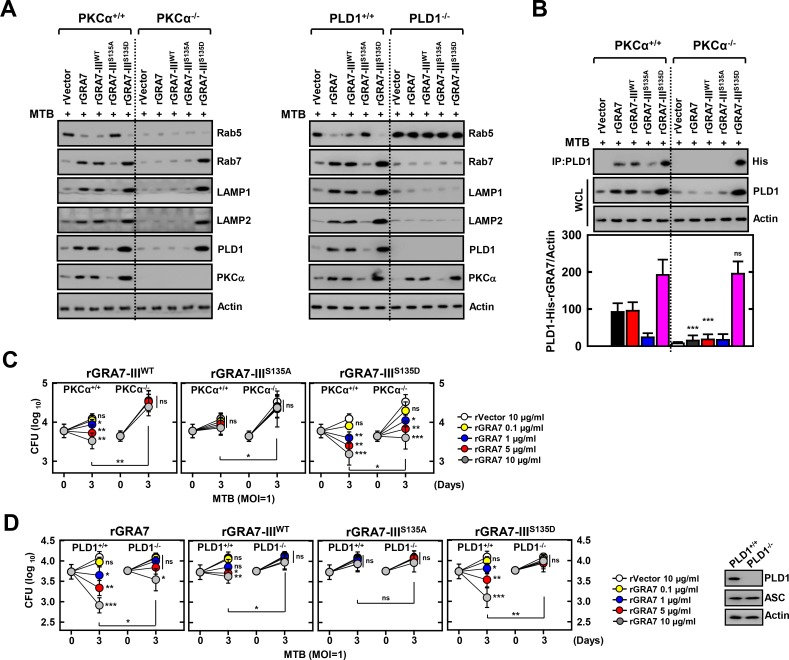
GRA7-III-induced activation of PLD1 and phagosomal maturation were required for antimicrobial activity in MTB-infected macrophages. (**A** and **B**) BMDMs were infected with MTB (MOI = 1) for 4 h and then stimulated with rGRA7 (5 μg/ml) and its mutants for 18 h. Mycobacteria-containing phagosome fractions were subsequently purified by sucrose-step-gradient-ultra-centrifugations, followed by IB to detect αRab5, αRab7, αLAMP1, αLAMP2, αPLD1, αPKCα, and αActin (**A**) or IP with αPLD1 and IB with αHis, αPLD1, and αActin (**B**). Quantitative analysis of the PLD1 interacts with His-rGRA7 in MTB-containing phagosomes band normalized to Actin is shown (lower). (**C** and **D**) Intracellular survival of MTB was assessed by CFU assay. BMDMs were infected with MTB for 4 h, followed by treatment with rGRA7, and then lysed to determine intracellular bacterial loads. (**D**, right) IB with αPLD1, αΑSC, and αActin in BMDMs. The data are representative of five independent experiments with similar results (**A** and **B**). Data shown are the mean ± SD of five experiments (**C** and **D**). Significant differences (*P < 0.05; **P < 0.01; ***P < 0.001) compared with PKCα+/+ and PKCα-/- (**B**) or rVector (**C** and **D**). CFU, colony-forming units. ns, not significant.

PLD1 activity regulates the actin cytoskeleton, vesicle trafficking for secretion and endocytosis, and receptor signaling. With the emerging concept of dynamic cycling of PLD1 inside the cell, some of the varying reports of localization may be due to differential rates and numbers of vesicles cycling in the cell lines used and thus differential regulation of PLD1 localization [14,15,28]. MTB preferentially infects alveolar macrophages, although mycobacteria allow only early endosome membrane fusion and induce phagosome arrest by selective Rab GTPase recruitment to avoid fusion with late endosomes and lysosomes [29,30]. To investigate the subcellular fractionation of PLD1, we treated MTB-infected BMDMs with rGRA7 and its mutants, and then examined the induction of protein levels of Rab5, Rab7, LAMP1, and LAMP2 regulators of phagosomal maturation in mycobacteria-containing phagosome fractions (phagosome and phago-lysosome) subsequently purified by sucrose-step-gradient-ultra-centrifugations. Interestingly, GRA7-induced MTB-containing phagosomes were recruited to late endosome and lysosome marker Rab7, LAMP1, and LAMP2, indicating that GRA7 facilitates mycobacterial phagosome-lysosome fusion in macrophages in a PKCα- and PLD1-dependent manner (Fig 6A). Furthermore, GRA7 associated with PLD1 in phagosomal fractions in a binding-dependent manner, and the phosphomimetic mutant (I^S135D^) of GRA7 markedly increased phagosomal trafficking and binding to PLD1, indicating that the constitutively active form (I^S135D^) of GRA7 ‘rescued’ PKCα deficiency (Fig 6B). Consistently, the viability and growth rate of intracellular MTB decreased following treatment with the phosphomimetic mutant (I^S135D^) in BMDMs from PKCα^+/+^ and PKCα^-/-^ mice, but not PLD1^-/-^ mice in dose-dependent manner (Fig 6C and 6D). These results collectively indicate that GRA7 facilitates phagosomal maturation through interactions with PLD1 and thereby, exerts marked control of bacterial killing activity against intracellular mycobacteria in a binding-dependent manner via PKCα.

### GRA7-I and-III-dependent host protective effects against MTB infection *in vivo*

**Fig 7:**
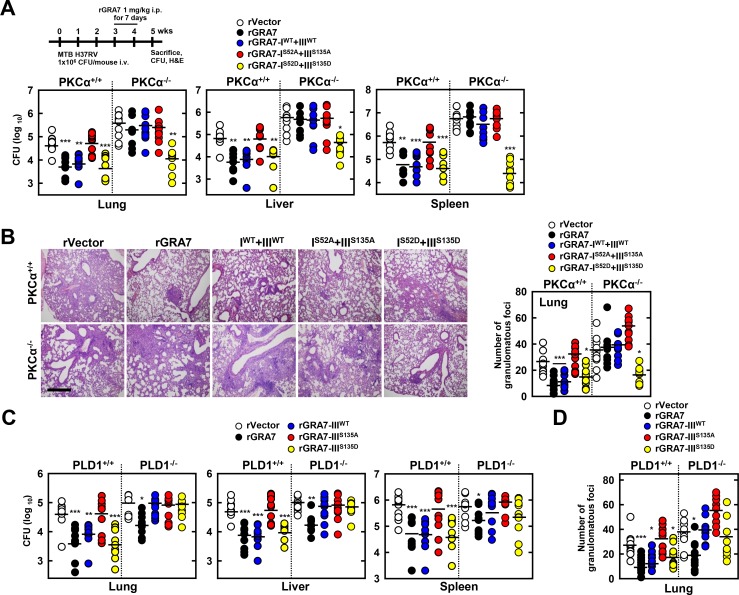
GRA7-I and -III showed anti-mycobacterial activity against MTB challenge *in vivo*. Schematic of the TB model treated with rGRA7 or vehicle (**A**, upper). (**A**) Bacterial loads in lung, liver, and spleen in PKCα^+/+^ and PKCα^-/-^ mice. *n* = 10. (**B**) Histopathology scores were obtained from H&E stained lung sections (left), as described in **Methods**. Number of granulomas observed in 10 different lung sections per mouse (right). Scale bar, 500 μm. (**C**) Bacterial loads in lung, liver, and spleen in PLD1^+/+^ and PLD1^-/-^ mice. *n* = 10. (**D**) Number of granulomas observed in 10 different lung sections per mouse. Black bars represent the median values. Student’s *t*-test and Grubbs’ outlier test were used for statistical analysis. The data are representative of two independent experiments with similar results. Significant differences (*P < 0.05; **P < 0.01; ***P < 0.001) compared with PKCα^+/+^ and PLD1^+/+^.

Drawing on the observation that GRA7-I and -III associate with ASC and PLD1, respectively, and which contributes to antimicrobial defense against MTB in macrophages (S7A Fig), we next evaluated the *in vivo* efficacy of rGRA7 and its binding mutants in a mouse model of established tuberculosis [31]. MTB-infected mice were given rGRA7 and its mutants, starting three weeks after infection. Mice treated with rGRA7-WT alone, rGRA7-I^WT^+III^WT^, or rGRA7-I^S53D^+III^S135D^, but not the binding deficient mutant (rGRA7-I^S53A^+III^S135A^) had significantly reduced bacillary load in the lung, liver, and spleen, and reduced formation of lung granulomatous lesions in size and number of foci, compared with vector-treated mice (Fig 7A and 7B). Notably, the phosphomimetic mutant (I^S53D^+III^S135D^) of rGRA7 drastically reduced bacillary load, at a level similar to rGRA7-WT in PKCα^+/+^ and PKCα^-/-^ mice.

We further investigated the effect of GRA7-III on *in vivo* host responses to MTB infection. As shown in Fig 7C and 7D, treatment with PLD1-binding domain (III^WT^) and the phosphomimetic mutant (III^S135D^) of rGRA7 markedly increased bacterial killing effects and number of granulomatous foci in PLD1^+/+^ mice, but not PLD1^-/-^ mice. Treatment with the PLD1-binding deficient mutant (III^S135A^) of rGRA7 had no significant effect on bacterial killing or granulomatous lesions in either PLD1^+/+^ or PLD1^-/-^ mice, indicated that the anti-mycobacterial effect of GRA7-III acts in a PLD1-binding dependent manner via PKCα *in vivo*. However, No significant difference was observed for inflammation score in lung (S7B and S7C Fig). The pharmacokinetics of therapeutic rGRA7 proteins were localized in alveolar macrophages was maintained for up to 7 days and gradually cleared until 25 days was studied by the fluorescence of the fluorophore Alexa 488-conjugated with the proteins (S8 Fig). These results unambiguously show that host defenses against MTB infection are substantially affected by GRA7-I and GRA7-III.

## Discussion

The central finding of this study is that the PKCα-mediated phosphorylation of *T*. *gondii* GRA7 is essential for the interaction between GRA7 and ASC or PLD1, which contributes to antimicrobial defense against MTB (S9 Fig). Specifically, we found that (1) PKCα specific phosphorylation of Ser52 and Ser135 of GRA7 *in vitro* and *in vivo* was functionally required for ASC and PLD1 interactions with GRA7, respectively, (2) GRA7 was a novel substrate of PKCα, (3) the N-terminal of GRA7 (GRA7-I) was sufficient for interaction with the PYD domain of ASC in mitochondria, leading to ASC oligomerization and inflammasome activation, and subsequent antimicrobial activity, (4) GRA7-III interacted with the PX domain of PLD1 in cytosol, facilitating its enzyme activity, phago-lysosomal biogenesis, and subsequent antimicrobial activity, (5) GRA7-I and -III-dependent host protective effects against MTB infection were demonstrated *in vivo*, and (6) a phosphomimetic mutant that constitutively activated GRA7 ‘rescued’ PKCα deficiency both *in vitro* and *in vivo*. Collectively, these observations indicate that *T*. *gondii* GRA7-mediated HDTs leading to an antimicrobial response, as a novel host defense mechanism may provide a unique opportunity for urgently needed therapeutic intervention strategies for TB and other infectious diseases.

Although it is well established that dense granule protein GRA7 is important for immunodiagnosis of toxoplasmosis in patients [32,33], new candidates for further effective vaccine development against *T*. *gondii* infection is the need [17,34,35]. Recent reports showed that GRA7 is associated with *T*. *gondii* ROP5 was required for efficient phosphorylation of Irga6 and additional component of the ROP5/ROP18 kinase complex [22,36] and binding of ROP2 and ROP4 was shown [37] in *T*. *gondii*. However, the modulation of host innate immunity by GRA7 in the early phases of infection is critical for the establishment of both the initial invasion and the subsequent maintenance of latent infection is have not been fully elucidated. Growing evidence suggests that host-pathogen interactions have led to the coevolution of toxoplasmosis-causing *T*. *gondii* with its host [17,22,38]. GRA7 binds to poly(rC) binding protein 1/PCBP1 along with PCBP2 and hnRNPK, corresponding to the principal cellular poly(rC) binding proteins according to yeast two-hybrid analysis. PCBP1 plays a part in the formation of a sequence-specific α-globin mRNP complex that is associated with the stability of α-globin mRNA [38]. Additionally, GRA7 directly binds to the active dimer of Irga6 in a GTP-dependent manner. The binding of GRA7 to Irga6 led to enhanced polymerization, rapid turnover, and eventual disassembly, which contributed to acute virulence in the mouse [22]. We recently showed that the GRA7-V (aa 201–236) domain led to physical and functional associations with TRAF6. Furthermore, GRA7-V-induced Th1 immune responses and protective efficacy were crucial for *T*. *gondii* infection *in vivo* [17]. In this study, we showed that host cell ASC, PLD1, and PKCα bind to GRA7. The GRA7 protein interacted with a number of host cell proteins including enzymes, and a broad spectrum of structural and functional subcellular organellar proteins revealing a new facet of the role of GRA7 in the regulation of innate host immune responses.

Our results correlate with those of previous studies showing that *T*. *gondii* is a novel activator of NLRP1 and NLRP3 inflammasomes by activating caspase-1, an enzyme that mediates cleavage and release of the proinflammatory cytokines IL-1β and IL-18 *in vitro* and *in vivo*, thereby establishing a role for these sensors in host resistance to toxoplasmosis [39–41]. Furthermore, Millholland *et al*. showed that a Gα subunit (Gα)q-coupled host-signaling cascade is required for the egress of *T*. *gondii*. Gαq-coupled signaling results in PKC-mediated loss of the host cytoskeletal protein adducin and weakening of the cellular cytoskeleton. This cytoskeletal compromise induces catastrophic Ca^2+^ influx mediated by the mechanosensitive cation channel TRPC6, which activates host calpain that in turn proteolyzes the host cytoskeleton allowing parasite release [42]. *T*. *gondii* induces prostaglandin E2 biosynthesis in macrophages by regulating arachidonic acid production through a Ca^2+^-dependent pathway and induction of cyclooxygenase-2 expression by a PKC-dependent pathway [43,44]. Reinforcing the feasibility of targeting host proteins as an antiparasitic strategy, mammalian PKC inhibitors demonstrate activity in murine models of toxoplasmosis. In this study, we focused on the role of GRA7-I and -III-dependent innate immunity. Future studies will aim to clarify the precise molecular mechanisms of GRA7 and GRA7-II and -IV-related signaling pathways in inflammatory responses and host defense.

HDTs aim to modulate immune responses in the TB lung [45,46]. Neutralization of pro-inflammatory cytokines such as IL-6, TNF-α, VEGF, and IFN-α/β, as well as anti-inflammatory IL-4, during severe pulmonary disease may help reduce ongoing parenchymal damage in the MTB-infected lung [27,45–47]. Alternatively, suboptimal activation of anti-TB immune responses due to regulatory T cell activity can be reversed by the use of the anti-cancer drug cyclophosphamide. Drugs with anti-TB potential, such as metformin, imatinib, ibuprofen, zileuton, valproic acid, and vorinostat as well as nutraceuticals such as 1,25D, may not only abate the bacterial burden via host-dependent mechanisms, but also fine-tune the immune response to MTB. These drugs increase phagocytosis of extracellular bacteria, improve emergency myeloid response, and increase autophagic and apoptotic killing of bacteria, subsequently editing the T cell response in favor of the host. Immune checkpoint inhibition with blockade of the PD-1/PD-1 ligand 1, CTLA-4/cytotoxic T lymphocyte-associated antigen 4, LAG3/lymphocyte-activation gene 3, and TIM3/T cell immunoglobulin pathways may improve the quality of the cellular immune response to MTB epitopes, as seen in cancer immunotherapy [4,5,45–47]. Our results partially correlate with those of previous studies showing that host-directed immunotherapy with clinically approved drugs that augment prostaglandin E2 level prevents acute mortality of MTB-infected mice. Thus, IL-1 and type I IFNs represent two major counter-regulatory classes of inflammatory cytokines that control the outcome of MTB infection and are functionally linked via eicosanoids [27], and IL-1β either directly or via enhancement by 1,25D promotes antimicrobial immunity against MTB infection [10,11]. Greco *et al*. showed that PKC-mediated Ca^2+^ mobilization, PLD activity, and (auto)phagolysosome maturation represent effector processes induced by apoptotic body-like liposomes carrying PA that concur with the intracellular killing of MTB [14]. The MTB-containing phagosomes is involved in arresting phagosome maturation and inhibiting phagolysosome biogenesis [6,8,9], however, rGRA7-induced PKCα regulates phagocytosis, PLD-dependent the biogenesis of phagolysosomes (Rab5 conversion to Rab7) by promoting the interaction of phagosomes with late endosomes and lysosomes, and Rab7 regulated phagosomal acidification, which is important for the killing of MTB in human macrophages [7,16]. Our current observations based on the study of GRA7-III co-localized with PLD1 and PKCα in the cytoplasm (Fig 5D and S6 Fig) have the proposal the localized on phagolysosomes, appearing as speckles and punctate spots, because of an artifact of rGRA7 overexpression. Further studies are needed to localization organelle population.

The rGRA7 have a function of biologicals as potential therapeutics. However, these rGRA7 do not fulfil the requirements of direct anti-mycobacterial agent, which represent feasible alternatives to conventional chemotherapy to TB, due to the still unclear specificity and selectivity does not enable linking the effects of rGRA7s to host immune systems, as well as limitation of animal experimental model, unknown off-target effects, pharmacokinetics, safety data, and their potential feasibility for *in vivo* proof-of-concept studies. Further analyses are required to find out whether rGRA7s can be translated to the *in vivo* situation or be observed in the presence of physiological condition to patient with TB.

In conclusion, we provide evidence of a critical role of PKCα-mediated phosphorylation of *T*. *gondii* GRA7 in the interaction between GRA7 and ASC or PLD1, which contributes to antimicrobial defense against MTB (S9 Fig). GRA7-I and -III-dependent host protective effects worked against MTB infection *in vivo*, and a phosphomimetic mutant that constitutively activated GRA7 ‘rescued’ PKCα deficiency. These observations reveal a new role for GRA7 in regulating innate immune responses in host protective immunity. Our findings establish proof of concept for HDT strategies that manipulate host GRA7-mediated immune networks. Further studies are needed to develop more effective GRA7-based potential therapeutic targets and to understand how GRA7 regulates host defense strategies against TB and other infectious diseases.

## Supporting Information

S1 Fig Identified of peptides by mass spectrometry analysis related to Fig 1A. (TIF)

S2 Fig GRA7 interaction with ASC and PLD1, but not PLD2, NLRP3, and NLRC4. (**A**) THP-1 cells were stimulated with rGRA7 (5 μg/ml) for 30 min., followed by IP with αPLD1, αTRAF6, or αASC and IB with αPLD1, αTRAF6, αASC, αHis, and αActin. (**B**) THP-1 cells were stimulated with rGRA7 for the indicated times, followed by IP with αHis-agarose bead and IB with αPLD2, αNLRP3, αNLRC4, αHis, and αActin. (**C**) THP-1 cells were pre-incubated with Cytochalasin D (Cyto D, 10 μM), phagocytosis inhibitor or solvent control for 30 min before treated with Alexa488-conjugated rGRA7 for the indicated times, followed by flow cytometry analysis to detect internalized Alexa488-rGRA7. The mean fluorescence intensity values of Alexa488-rGRA7 from flow cytometry were used to generate phagocytosis rate kinetics (bottom). (**D**) THP-1 cells were pre-incubated with Cytochalasin D (Cyto D, 5, 10, 20 μM) and stimulated with rGRA7 for the indicated times, followed by IP with αHis-agarose bead and IB with αPLD1, αTRAF6, αASC, αHis, and αActin. (**E**) Schematic diagram of the structures of PLD1 and its mutants. The data are representative of three independent experiments with similar results (**A**—**D**). SC, solvent control (0.1% DMSO).
(TIF)

S3 Fig GRA7 interaction with ASC and PLD1 was dependent on PKCα. (**A**) Phos-tag and SDS-PAGE analysis of GST-GRA7 expressed together with Flag-tagged PKCβ, PKCδ, or PKCξ in 293T cells left untreated (CIP-) or treated calf intestinal alkaline phosphatase (CIP+), and subjected to GST pulldown, followed by IB with αGST. WCLs were used for IB with αFlag or αActin. (**B**) At 48 hr post-transfection with mammalian GST, GST-GRA7, or GST-GRA7-V constructs together with V5-ASC or AU1-PLD1, 293T cells were used for GST pulldown, followed by IB with αV5 and αAU1. WCLs were used for IB with αGST, αV5, αAU1, αPKCα or αActin. (**C**) At 48 hr transduction with lentivirus-shRNA-NS or lentivirus-shRNA-PKCα (MOI = 50), THP-1 cells were stimulated with rGRA7 (5 μg/ml) for 30 min., followed by IP with αHis-agarose bead and IB with αASC, αPLD1, αPKCα, and αActin. (**D**) THP-1 cells were pre-incubated with PKCα (C2-4) (5, 10, 20 μM) and stimulated with rGRA7 for 30 min., followed by IP with αHis-agarose bead and IB with αPLD1, αASC, αPKCα, and αActin. The data are representative of three independent experiments with similar results (**A**—**D**).
(TIF)

S4 Fig GRA7-I activate inflammasomes in mitochondria in ASC-binding dependent manner. (**A**) BMDMs from PKCα^+/+^ and PKCα^-/-^ were stimulated with rGRA7 (5 μg/ml) and its mutants for the indicated times and culture supernatants were harvested and analyzed for cytokine ELISA for TNF-α and IL-6. (**B**) BMDMs was transduced with lentivirus-shRNA-NS or lentivirus-shRNA-ASC (MOI = 100) with polybrene (8 μg/mL) (right) for 2 days, followed by IB with αASC, αPLD1, and αActin. (**C**) The number of ASC pyroptosome was counted using a fluorescent microscope and the ASC speck-containing cells were represented as a relative percentage compared to the total cell number related to **Fig 3E**. (**D**) BMDMs from PKCα^+/+^ and PKCα^-/-^ were stimulated with rGRA7-I and its mutants for 18 h. The cells were then subcellularly fractionated, subjected to co-IP with αHis, followed by IB analysis with αASC and αPKCα. Levels of tubulin (cytosolic), calnexin (endoplasmic reticulum (ER) and mitochondria-associated membrane (MAM)), fatty acid CoA ligase 4 (FACL4, MAM) and voltage-dependent anion channels (VDAC, mitochondrial) protein in each fraction were determined by IB analysis. Data shown are the means ± SD of five experiments (**A** and **C**). The data are representative of three independent experiments with similar results (**B** and **D**).
(TIF)

S5 Fig rGRA7-I’s effect on mycobacteria growth. *M*. *tuberculosis* H37Rv were cultures in 7H9 broth contained 10% OADC in presence of rVector, rGRA7-WT, -I, or -III (10 μg/ml) for the indicated times at 37°C. Measure the OD_600_ every 3 days. Data shown are the means ± SD of three experiments.
(TIF)

S6 Fig GRA7-III interacts with PLD1 in cytosol. (**A**) The co-localization index (%) between PLD1 and GRA7 were quantified and validated statistically by Pearson coefficient, as specified by the ZEN 2009 software, related to **Fig 5D**. Data shown are the means ± SD of five experiments. (**B**) BMDMs from PKCα^+/+^ and PKCα^-/-^ were stimulated with rGRA7-III and its mutants for 18 h. The cells were then subcellularly fractionated, subjected to co-IP with αHis, followed by IB analysis with αPLD1 and αPKCα. Levels of tubulin (cytosolic), calnexin (endoplasmic reticulum (ER) and mitochondria-associated membrane (MAM)), fatty acid CoA ligase 4 (FACL4, MAM) and voltage-dependent anion channels (VDAC, mitochondrial) protein in each fraction were determined by IB analysis. The data are representative of three independent experiments with similar results.
(TIF)

S7 Fig Effects of GRA7-induced anti-mycobacterial activity and inflammation score *in vivo*. (**A**) Intracellular survival of MTB was assessed by CFU assay. BMDMs were infected with MTB for 4 h, followed by treatment with rGRA7, and then lysed to determine intracellular bacterial loads. Data shown are the mean ± SD of five experiments. Significant differences (**P < 0.01; ***P < 0.001) compared with rVector. (**B** and **C**) Whole lung photo (**B**, left, related to **Fig 7B**) and immunopathology scores were obtained from H&E stained lung sections (**B**, right and **C**), as described in **Methods**. The data are representative of three independent experiments with similar results (**B**, left, 12.5 X). *n* = 10 (**B**, right and **C**). CFU, colony-forming units. ns, not significant.
(TIF)

S8 Fig Therapeutic rGRA7 proteins are uptaken by cells of the reticuloendothelial system in the lung. Schematic of the pharmacokinetic analysis in TB model treated with rGRA7 (upper). Mycobacteria-infected mice were injected with Alexa488-conjugated proteins for 7 consecutive days and then lung was harvested at indicated time points and immune-stained with αMac-3 or DAPI. Pharmacokinetic analysis of proteins in the lung was visualized through a multi-photon confocal laser scanning microscope system. The data are representative of three independent experiments with similar results. Scale bar, 10 μm.
(TIF)

S9 Fig Schematic model for the roles of GRA7 and GRA7-mediated regulatory pathways against intracellular pathogens such as *Mycobacteria* and *T*. *gondii*. Please see the Discussion for detail.
(TIF)

S1 Text (DOC)
