# Supplementary figures and images for: Toxoplasma gondii GRA7-Targeted ASC and PLD1 Promote Antibacterial Host Defense via PKCα
Source: PLoS Pathog. 2017 Jan 26;13(1):e1006126. doi: 10.1371/journal.ppat.1006126 (PMC5268361; doi:10.1371/journal.ppat.1006126)

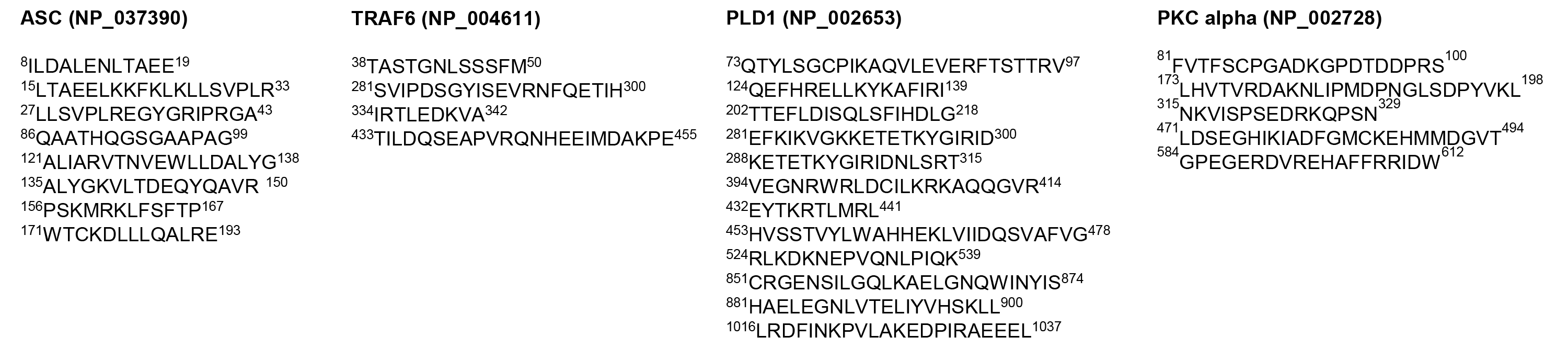

Supplement: S1 Fig — (TIF) [file ppat.1006126.s001.tif]

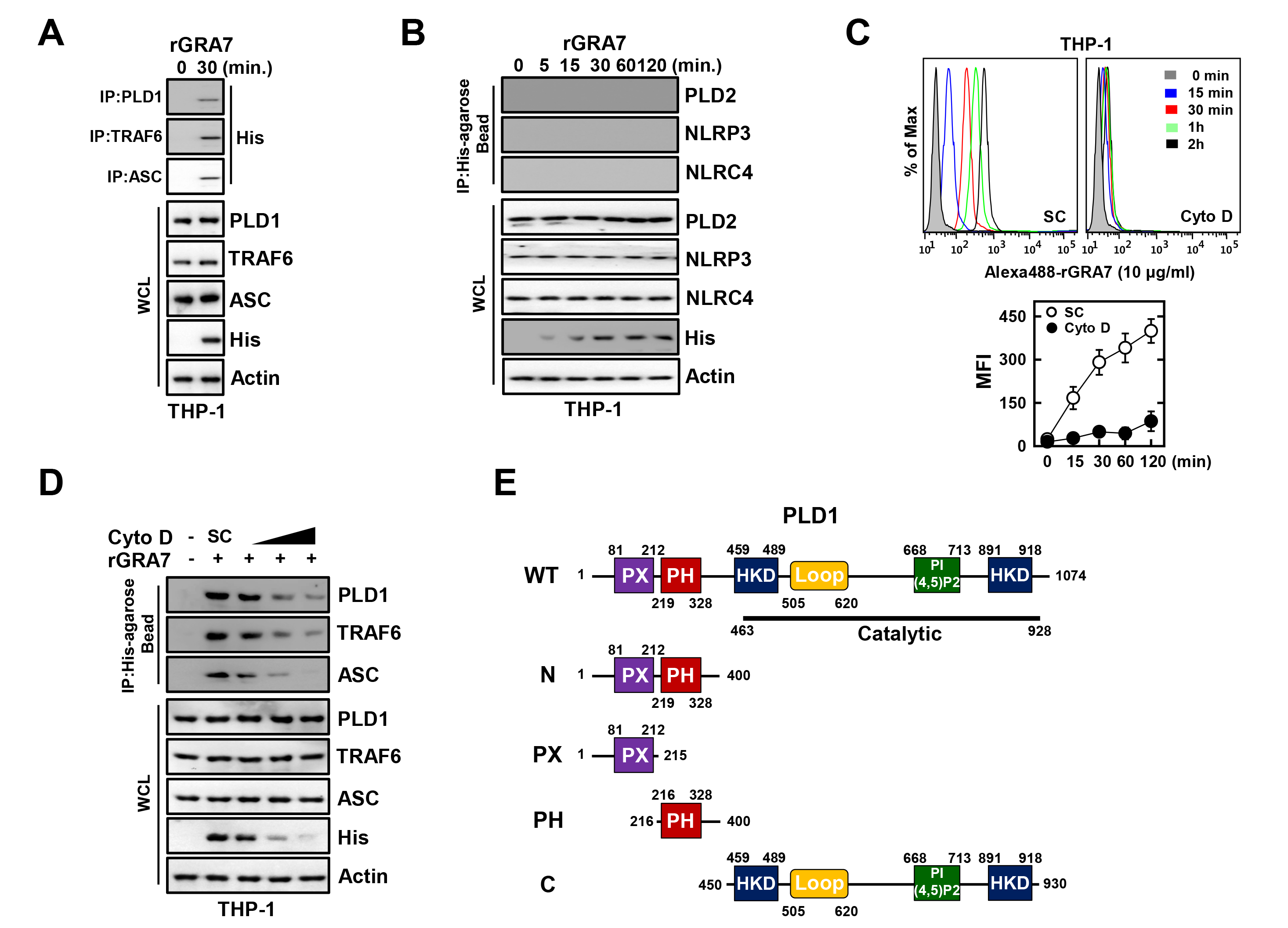

Supplement: S2 Fig — (A) THP-1 cells were stimulated with rGRA7 (5 μg/ml) for 30 min., followed by IP with αPLD1, αTRAF6, or αASC and IB with αPLD1, αTRAF6, αASC, αHis, and αActin. (B) THP-1 cells were stimulated with rGRA7 for the indicated times, followed by IP with αHis-agarose bead and IB with αPLD2, αNLRP3, αNLRC4, αHis, and αActin. (C) THP-1 cells were pre-incubated with Cytochalasin D (Cyto D, 10 μM), phagocytosis inhibitor or solvent control for 30 min before treated with Alexa488-conjugated rGRA7 for the indicated times, followed by flow cytometry analysis to detect internalized Alexa488-rGRA7. The mean fluorescence intensity values of Alexa488-rGRA7 from flow cytometry were used to generate phagocytosis rate kinetics (bottom). (D) THP-1 cells were pre-incubated with Cytochalasin D (Cyto D, 5, 10, 20 μM) and stimulated with rGRA7 for the indicated times, followed by IP with αHis-agarose bead and IB with αPLD1, αTRAF6, αASC, αHis, and αActin. (E) Schematic diagram of the structures of PLD1 and its mutants. The data are representative of three independent experiments with similar results (A—D). SC, solvent control (0.1% DMSO). (TIF) [file ppat.1006126.s002.tif]

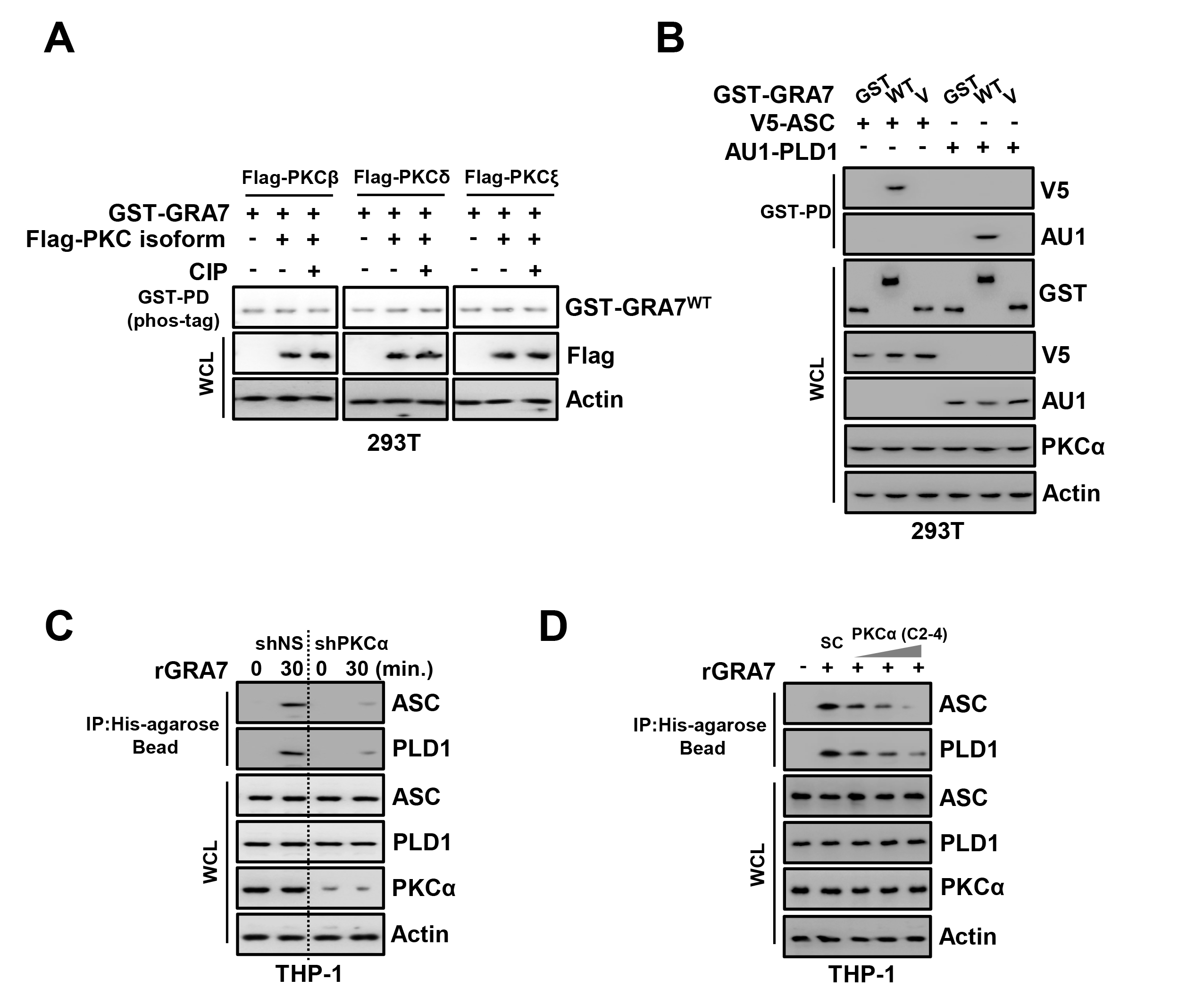

Supplement: S3 Fig — (A) Phos-tag and SDS-PAGE analysis of GST-GRA7 expressed together with Flag-tagged PKCβ, PKCδ, or PKCξ in 293T cells left untreated (CIP-) or treated calf intestinal alkaline phosphatase (CIP+), and subjected to GST pulldown, followed by IB with αGST. WCLs were used for IB with αFlag or αActin. (B) At 48 hr post-transfection with mammalian GST, GST-GRA7, or GST-GRA7-V constructs together with V5-ASC or AU1-PLD1, 293T cells were used for GST pulldown, followed by IB with αV5 and αAU1. WCLs were used for IB with αGST, αV5, αAU1, αPKCα or αActin. (C) At 48 hr transduction with lentivirus-shRNA-NS or lentivirus-shRNA-PKCα (MOI = 50), THP-1 cells were stimulated with rGRA7 (5 μg/ml) for 30 min., followed by IP with αHis-agarose bead and IB with αASC, αPLD1, αPKCα, and αActin. (D) THP-1 cells were pre-incubated with PKCα (C2-4) (5, 10, 20 μM) and stimulated with rGRA7 for 30 min., followed by IP with αHis-agarose bead and IB with αPLD1, αASC, αPKCα, and αActin. The data are representative of three independent experiments with similar results (A—D). (TIF) [file ppat.1006126.s003.tif]

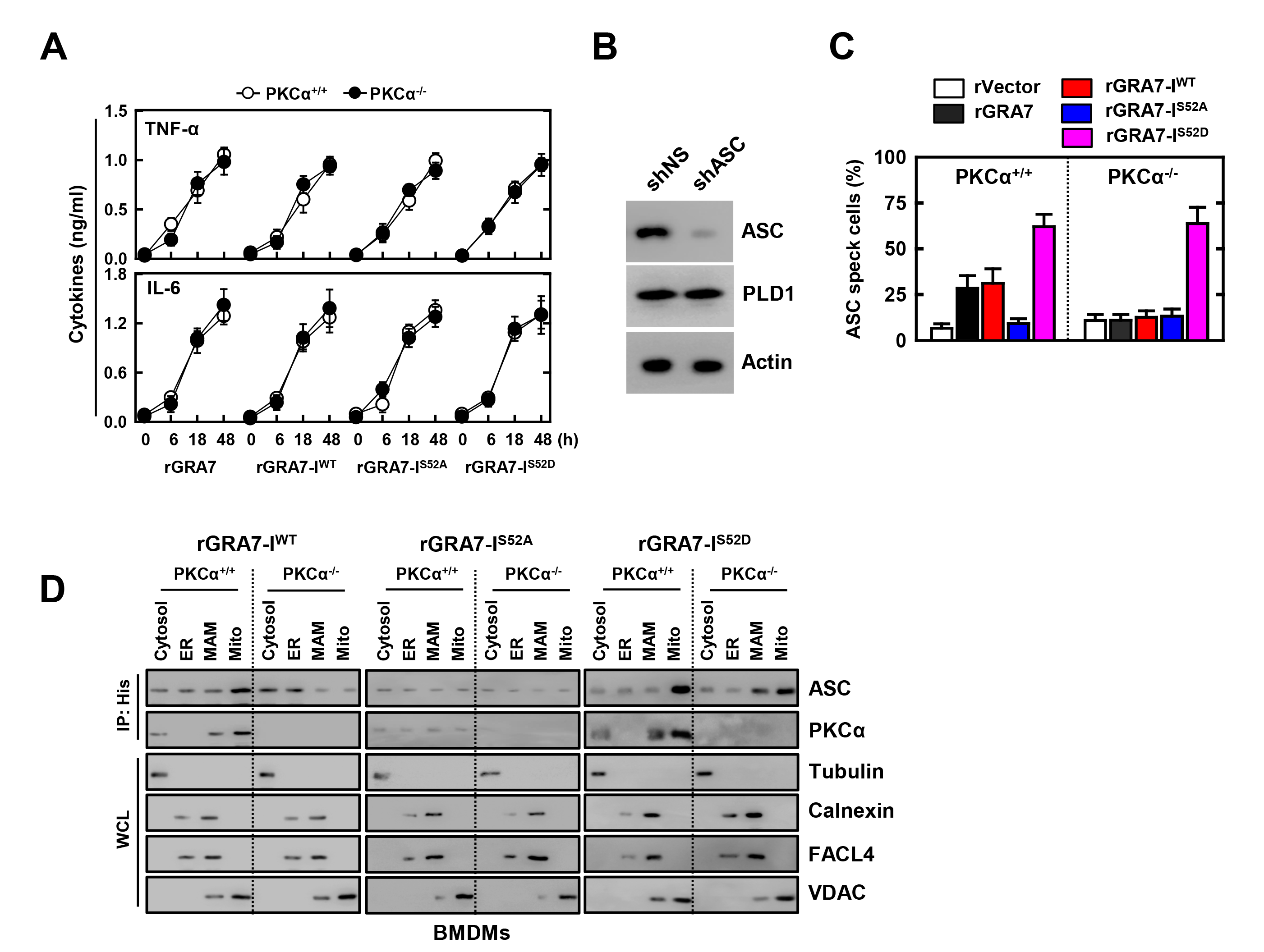

Supplement: S4 Fig — (A) BMDMs from PKCα+/+ and PKCα-/- were stimulated with rGRA7 (5 μg/ml) and its mutants for the indicated times and culture supernatants were harvested and analyzed for cytokine ELISA for TNF-α and IL-6. (B) BMDMs was transduced with lentivirus-shRNA-NS or lentivirus-shRNA-ASC (MOI = 100) with polybrene (8 μg/mL) (right) for 2 days, followed by IB with αASC, αPLD1, and αActin. (C) The number of ASC pyroptosome was counted using a fluorescent microscope and the ASC speck-containing cells were represented as a relative percentage compared to the total cell number related to Fig 3E. (D) BMDMs from PKCα+/+ and PKCα-/- were stimulated with rGRA7-I and its mutants for 18 h. The cells were then subcellularly fractionated, subjected to co-IP with αHis, followed by IB analysis with αASC and αPKCα. Levels of tubulin (cytosolic), calnexin (endoplasmic reticulum (ER) and mitochondria-associated membrane (MAM)), fatty acid CoA ligase 4 (FACL4, MAM) and voltage-dependent anion channels (VDAC, mitochondrial) protein in each fraction were determined by IB analysis. Data shown are the means ± SD of five experiments (A and C). The data are representative of three independent experiments with similar results (B and D). (TIF) [file ppat.1006126.s004.tif]

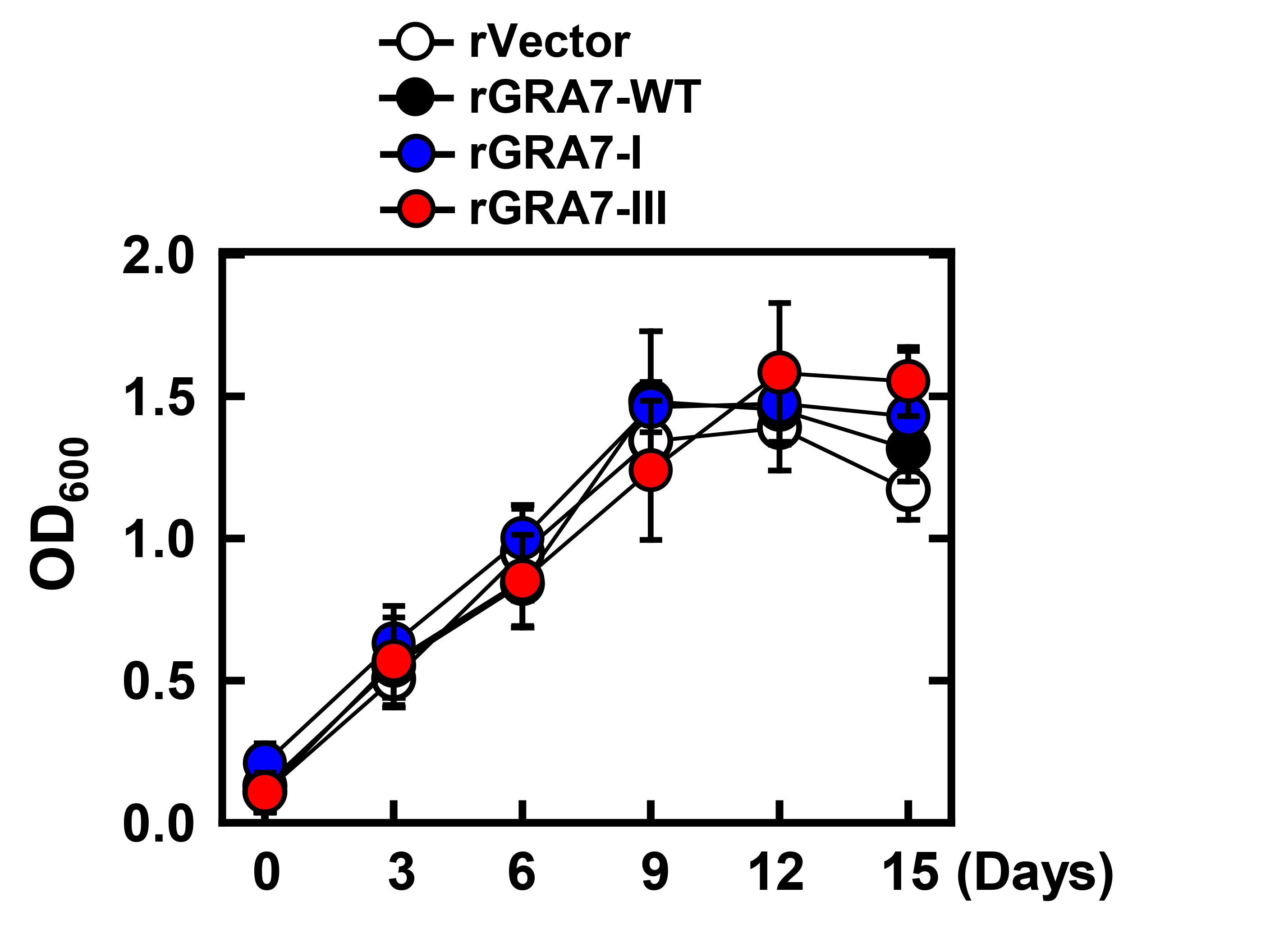

Supplement: S5 Fig — M. tuberculosis H37Rv were cultures in 7H9 broth contained 10% OADC in presence of rVector, rGRA7-WT, -I, or -III (10 μg/ml) for the indicated times at 37°C. Measure the OD600 every 3 days. Data shown are the means ± SD of three experiments. (TIF) [file ppat.1006126.s005.tif]

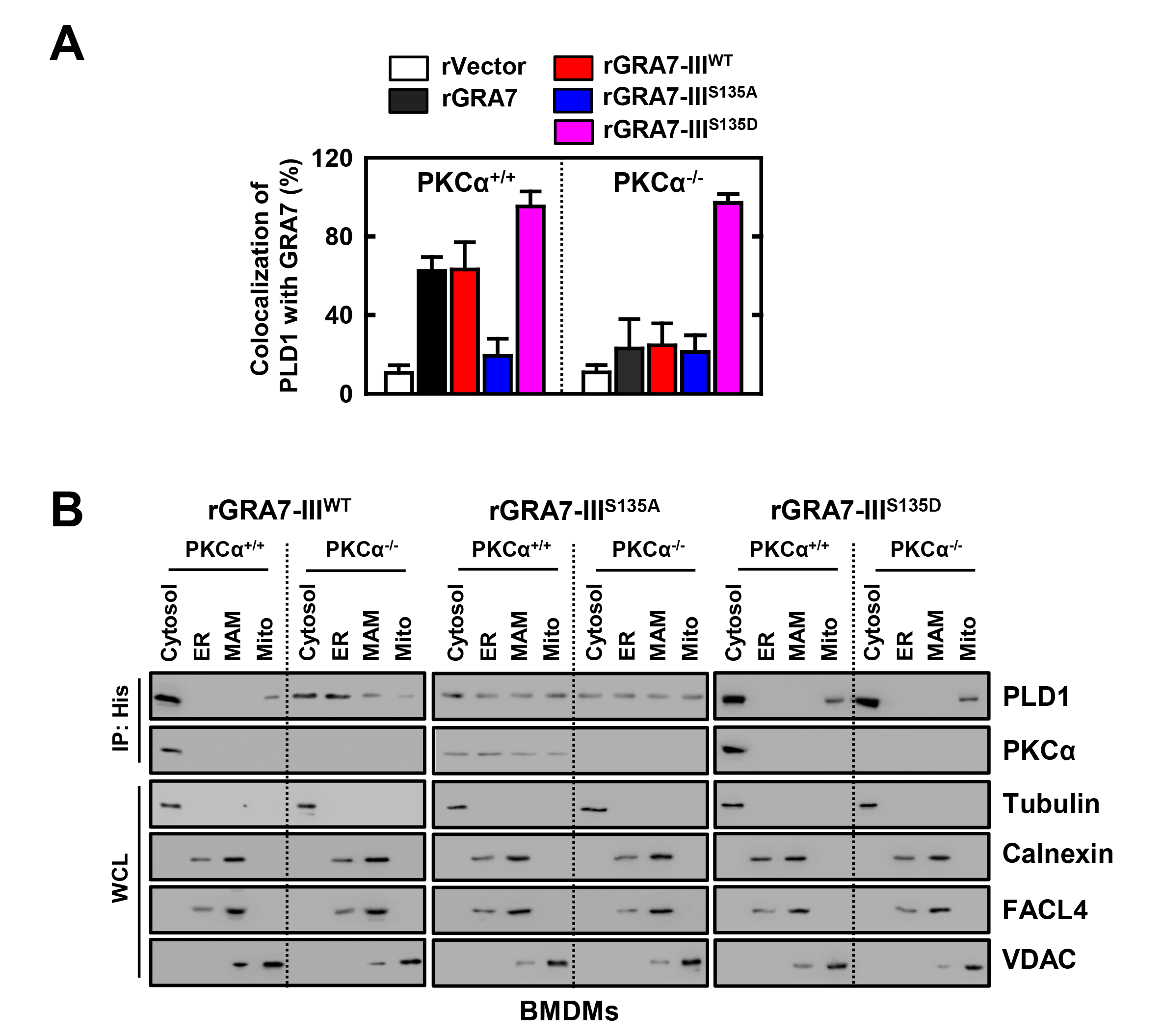

Supplement: S6 Fig — (A) The co-localization index (%) between PLD1 and GRA7 were quantified and validated statistically by Pearson coefficient, as specified by the ZEN 2009 software, related to Fig 5D. Data shown are the means ± SD of five experiments. (B) BMDMs from PKCα+/+ and PKCα-/- were stimulated with rGRA7-III and its mutants for 18 h. The cells were then subcellularly fractionated, subjected to co-IP with αHis, followed by IB analysis with αPLD1 and αPKCα. Levels of tubulin (cytosolic), calnexin (endoplasmic reticulum (ER) and mitochondria-associated membrane (MAM)), fatty acid CoA ligase 4 (FACL4, MAM) and voltage-dependent anion channels (VDAC, mitochondrial) protein in each fraction were determined by IB analysis. The data are representative of three independent experiments with similar results. (TIF) [file ppat.1006126.s006.tif]

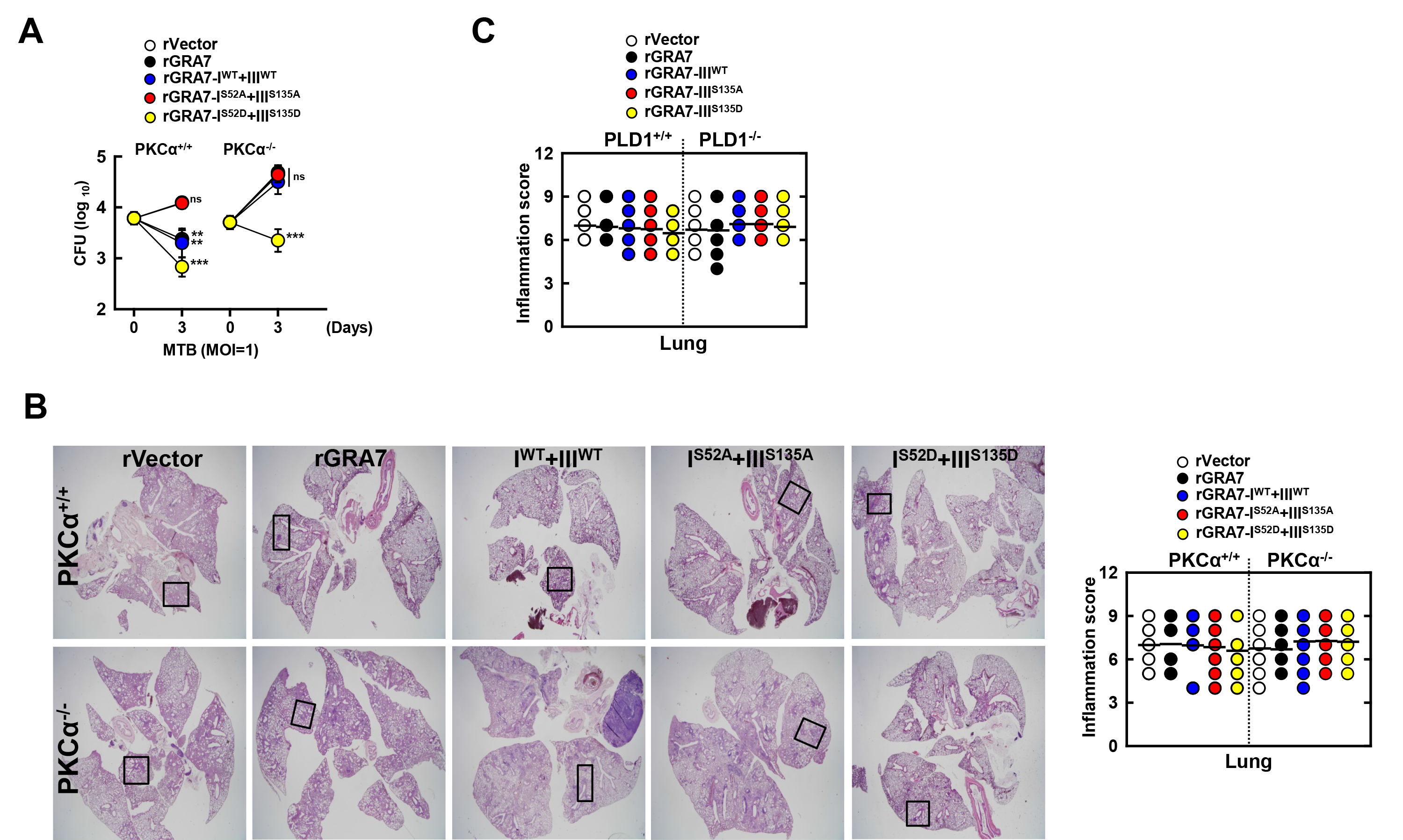

Supplement: S7 Fig — (A) Intracellular survival of MTB was assessed by CFU assay. BMDMs were infected with MTB for 4 h, followed by treatment with rGRA7, and then lysed to determine intracellular bacterial loads. Data shown are the mean ± SD of five experiments. Significant differences (**P < 0.01; ***P < 0.001) compared with rVector. (B and C) Whole lung photo (B, left, related to Fig 7B) and immunopathology scores were obtained from H&E stained lung sections (B, right and C), as described in Methods. The data are representative of three independent experiments with similar results (B, left, 12.5 X). n = 10 (B, right and C). CFU, colony-forming units. ns, not significant. (TIF) [file ppat.1006126.s007.tif]

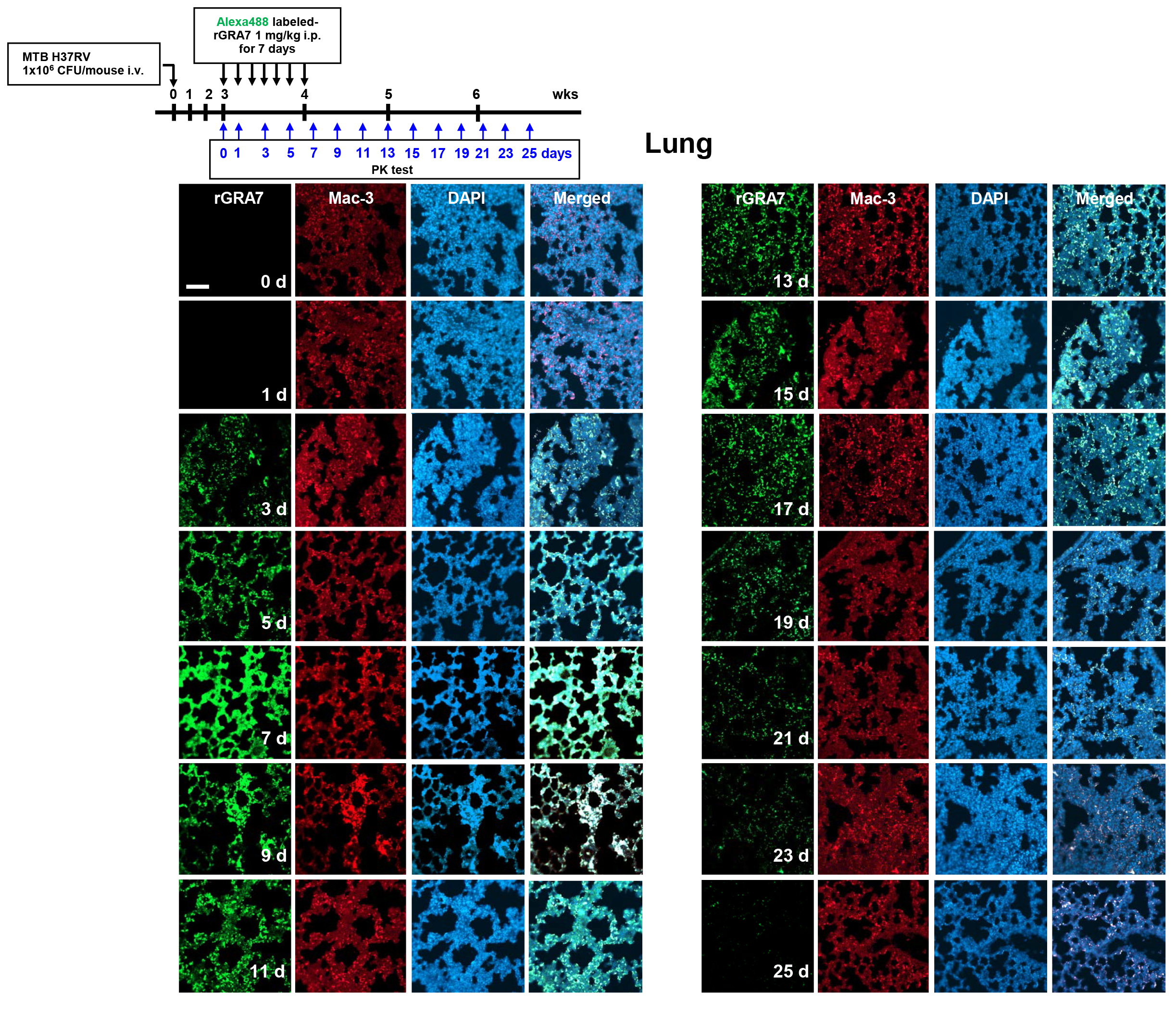

Supplement: S8 Fig — Schematic of the pharmacokinetic analysis in TB model treated with rGRA7 (upper). Mycobacteria-infected mice were injected with Alexa488-conjugated proteins for 7 consecutive days and then lung was harvested at indicated time points and immune-stained with αMac-3 or DAPI. Pharmacokinetic analysis of proteins in the lung was visualized through a multi-photon confocal laser scanning microscope system. The data are representative of three independent experiments with similar results. Scale bar, 10 μm. (TIF) [file ppat.1006126.s008.tif]

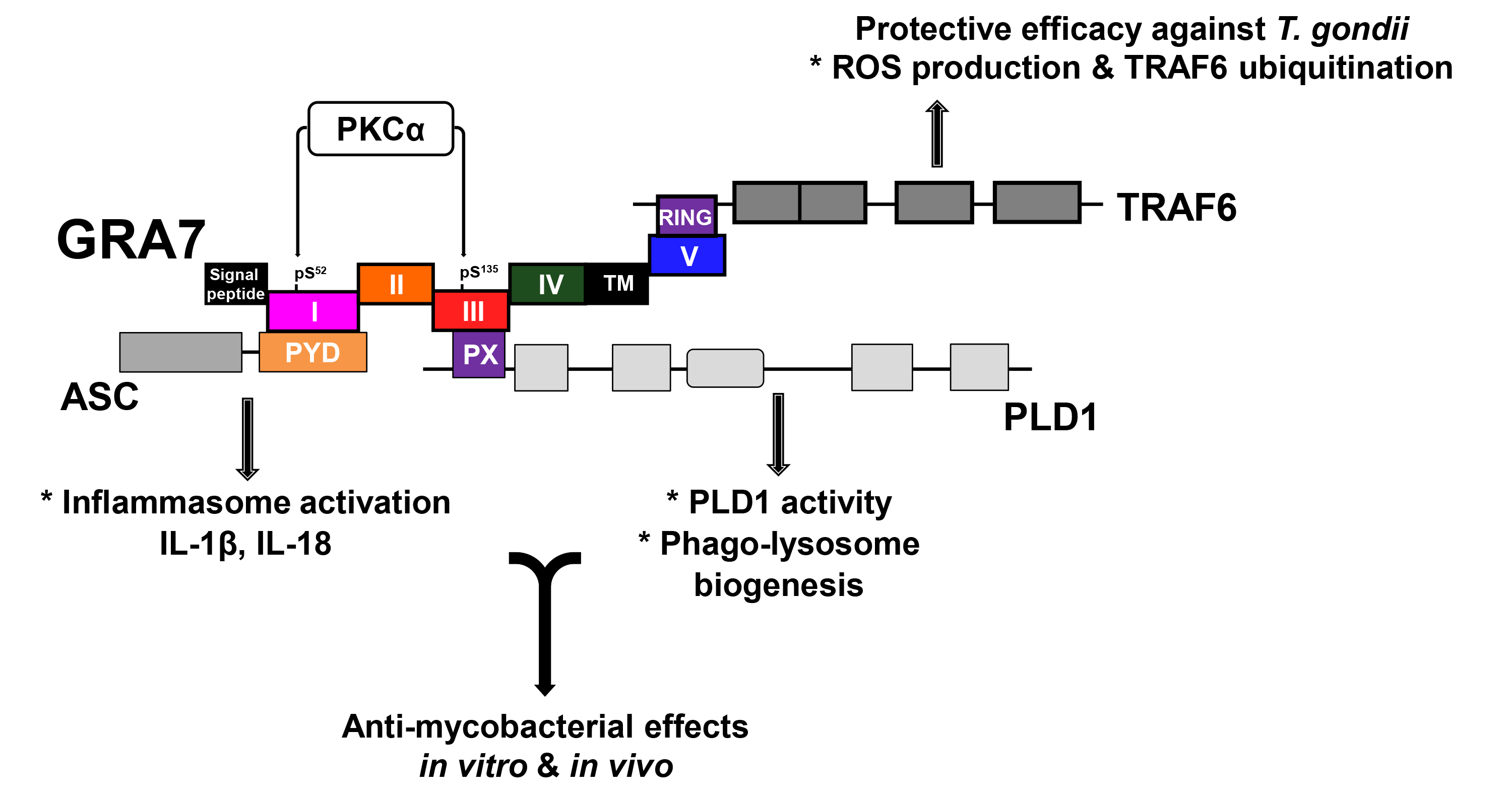

Supplement: S9 Fig — Please see the Discussion for detail. (TIF) [file ppat.1006126.s009.tif]
