# Supplementary material for: Toxoplasma gondii GRA7-Targeted ASC and PLD1 Promote Antibacterial Host Defense via PKCα
Source: PLoS Pathog. 2017 Jan 26;13(1):e1006126. doi: 10.1371/journal.ppat.1006126 (PMC5268361; doi:10.1371/journal.ppat.1006126)
Supplement: S1 Text — (DOC) [file ppat.1006126.s010.doc]

**Supplemental Information**

**Supplemental Experimental Procedures**

*Protein purification and mass spectrometry*

Precipitates were washed extensively with lysis buffer. Proteins bound to beads were eluted and separated on a NuPAGE 4–12% Bis-Tris gradient gel (Life Technologies). After silver staining (Life Technologies), specific protein bands were excised and analysed by ion-trap mass spectrometry at the Korea Research Institute of Bioscience and Biotechnology Mass Spectrometry facility, and amino acid sequences were determined by tandem mass spectrometry and database searches

*Immunoblot analysis and Immunoprecipitation*

For Western blot analysis, primary Abs were used at a 1/1,000 dilution. For the immunoprecipitation, cells were harvested and lysed in NP-40 buffer supplemented with a complete protease inhibitor cocktail (Roche). The lysates were mixed and precipitated with Abs and protein A-Sepharose by incubation at 4°C for 18 h on a rotator. For GST Pulldown assays, pre-cleared lysates were mixed with 50% slurry of glutathione-conjugated Sepharose beads (Amersham Biosciences), and the binding reactionwas incubated for 4 h at 4°C. The samples were subsequently solubilized in SDS sample buffer and separated by SDS-PAGE for Western blot analysis. Antibody binding was visualized by chemiluminescence (ECL;Millipore) and detected by a Vilber chemiluminescence analyzer (Fusion SL 3;Vilber Lourmat).

*Cellular fractionation*

Cytosol and mitochondria were isolated from cells using a Mitochondria Fractionation Kit (Active Motif, 40015) or as described previously . Cytosol, microsomes (endoplasmic reticulum, ER), mitochondria-associated membrane (MAM) fraction and pure mitochondria were isolated from cells using an Endoplasmic Reticulum Isolation Kit (Sigma, ER0100) or as described previously . Subcellular fractionated proteins were lysed in buffer containing 2% SDS and boiled with 2x reducing sample buffer for SDS-PAGE.

*Isolation of MTB-containing phagosomes*

Eight 15-cm^2^ plates of BMDMs were used for each condition, bacteria at an MOI of 10 were infected to macrophages for 2 h, washed and then rGRA7 stimulated for 2 h. Infected cells were collected, lysed and subjected to fractionation as described previously . The phagosomal fractions were extracted by the cell lysis buffer containing 25 mM Tris–HCl pH 7.6, 150 mM NaCl, 1% Nonidet P-40, 1% sodium deoxycholate and 0.1% SDS. We confirmed that mycobacterial proteins are not extracted by the cell lysis buffer as described previously . For immunoblotting analysis, aliquots of 6 μg of phagosomal fractions were separated by SDS–PAGE and then subjected to immunoblotting analysis

*CFU assay*

To assay bacterial viability within macrophages, cells were infected with MTB for 4 h and then washed with PBS to remove extracellular bacteria. Thereafter, the infected cells were incubated for the indicated time periods. Finally, cells were harvested and lysed with 0.3% saponin (Sigma-Aldrich) to release the intracellular bacteria, and cell lysates were then resuspended vigorously, transferred to screw cap tubes, and sonicated in a preheated 37°C water bath sonicator (Elma) for 5 min. Aliquots of the sonicates were then diluted 10-fold in Middlebrook 7H9 medium. Four dilutions of each sample were plated separately on Middlebrook 7H10 agar plates and incubated at 37°C with for 2–3 weeks

*Measurements of cytokines*

Mouse cytokines in culture supernatants were measured by a BD OptEIA ELISA set (BD Pharmingen) as described previously . All assays were performed as recommended by the manufacturers.

*Lentiviral shRNA production and transduction*

Lentiviral shRNA *production, concentration,* titration *and transduction* were described previously using the target shRNA plasmid DNA (human PKCα and mouse ASC) were purchased from Open Biosystems. A parallel experiment using a GFP-encoding lentivirus (the pGIPZ lentiviral vector; Open Biosystems) indicated that 80% of cells were successfully transduced by the virus.

*Immunostaining of ASC pyroptosomes*

Cells were fixed with 4% paraformaldehyde and 0.1% NP40, washed and stained with anti-Asc antibody and FITC-conjugated anti–rat antibody (Sigma) as a secondary antibody. Imaging analysis was performed using laser-scanning confocal microscopy (model LSM 800; Zeiss), and percentage of cells containing ASC pyroptosomes was determined by counting at least 300 cells in 5 separate fields.

*ASC oligomerization assay*
Macrophages were lysed with TBS buffer (50 mM Tris-HCl (pH 7.4) and 150 mM NaCl) containing 0.5% Triton X-100, EDTA-free protease inhibitor ‘cocktail’ and phosphatase inhibitor cock­tail (Roche). The lysates were centrifuged at 6,000*g* for 15 min at 4 °C, and the pellets and supernatants were used as the Triton-insoluble fractions and Triton-soluble fractions, respectively. For the detection of ASC oligomeriza­tion, the Triton-insoluble pellets were washed twice with TBS buffer and then were resuspended in 300 μl TBS buffer. The resuspended pellets underwent crosslinkage for 30 min at 37 °C with 2 mM disuccinimidyl suberate (Pierce) and then were centrifuged for 15 min at 6,000*g*. The pellets were dissolved in SDS sample buffer.

*Peptide spot arrays*

The peptide membrane was blocked at RT for 30 minutes in binding buffer containing 5% BSA. Recombinant PKCα (5 nM) was added to 50 mM HEPES, pH 7.4, 100 mM NaCl, 10 mM MgCl_2_, 100 mΜ ATP, 1 mM CaCl2, 6 μCi/ml [γ-32P]ATP and incubated at RT for 15 minutes. The membrane was washed three times with 100 mM sodium phosphate pH 7.0, 1 M NaCl, 10 mM EDTA and visualized using phosphorimaging (Fuji phosphor imager). The phosphorylation of each peptide was detected and quantified using Multi Gauge version 3.0 (Fujifilm).

*Recombinant GRA7 protein*

The rGRA7 mutant proteins were dialyzed using permeable cellulose membrane and tested for lipopolysaccharide contamination by a *Limulus* amebocyte lysate assay (BioWhittaker), and contained a less than 20 pg/ml with the concentrations of the rGRA7 protein used in the experiments.

*Lung Histopathology*

Serial sections (4 μm) were stained with hematoxylin and eosin (H&E).. A semiquantitative histopathologic scoring system was developed on the basis of the presence and abundance of the following: (*1*) perivascular edema (0, absent; 1, mild to moderate, involving fewer than 25% of the perivascular spaces; 2, moderate to severe, involving more than 25% but less than 75% of perivascular spaces; or 3, severe, involving more than 75% of perivascular spaces); (*2*) perivascular/peribronchial acute inflammation (0, absent; 1, mild acute inflammation in the perivascular edematous space, with fewer than 5 neutrophils per high-power field [hpf]; 2, moderate acute inflammation in the perivascular spaces, extending to involve the peribronchial spaces, with more than 5 neutrophils per hpf in these regions; or 3, severe, acute inflammation in the perivascular and peribronchial spaces with numerous neutrophils encircling most [50%] of bronchioles); (*3*) goblet-cell metaplasia of bronchioles (0, absent; 1, few goblet cells present in one or two bronchiolar profiles; or 2, large numbers of goblet cells present); and (*4*) eosinophilic macrophages in alveolar spaces (0, absent; 1, present in fewer than 25% of alveolar spaces; or 2, present in 25% of alveolar spaces). A total inflammatory score (range 0 to 10), taken as the sum of the individual scores, was determined by a pulmonary pathologist who was blinded to genotype and treatment-group assignment.

*Pharmacokinetic analysis of proteins*

To measure the cellular uptake quantitatively, Alexa488 was conjugated to the rGRA7 proteins according to manufacturer's protocol ([Alexa Fluor® 488 Protein Labeling Kit, Thermo Fisher Scientific](https://tools.thermofisher.com/content/sfs/manuals/mp10235.pdf)). The Alexa488-conjugated proteins were intraperitoneally administrated for 7 consecutive days. At indicated time points, lung was harvested and fixed in 10% buffered formalin and embedded in paraffin. Fresh 4-μm frozen sections were stained with Mac-3 (Alexa Fluor 568[)](https://www.thermofisher.com/order/catalog/product/A20184) for alveolar macrophages and DAPI for nuclear counterstains, as previously described . Lung samples were visualized through a laser-scanning confocal microscopy (model LSM 800; Zeiss).

**Supplemental References**

1. Yang CS, Kim JJ, Kim TS, Lee PY, Kim SY, et al. (2015) Small heterodimer partner interacts with NLRP3 and negatively regulates activation of the NLRP3 inflammasome. Nat Commun 6: 6115.

2. Bozidis P, Williamson CD, Colberg-Poley AM (2007) Isolation of endoplasmic reticulum, mitochondria, and mitochondria-associated membrane fractions from transfected cells and from human cytomegalovirus-infected primary fibroblasts. Curr Protoc Cell Biol Chapter 3: Unit 3 27.

3. Wieckowski MR, Giorgi C, Lebiedzinska M, Duszynski J, Pinton P (2009) Isolation of mitochondria-associated membranes and mitochondria from animal tissues and cells. Nat Protoc 4: 1582-1590.

4. Beatty WL, Rhoades ER, Hsu DK, Liu FT, Russell DG (2002) Association of a macrophage galactoside-binding protein with Mycobacterium-containing phagosomes. Cell Microbiol 4: 167-176.

5. Lee BY, Jethwaney D, Schilling B, Clemens DL, Gibson BW, et al. (2010) The Mycobacterium bovis bacille Calmette-Guerin phagosome proteome. Mol Cell Proteomics 9: 32-53.

6. Yang CS, Lee JS, Rodgers M, Min CK, Lee JY, et al. (2012) Autophagy protein Rubicon mediates phagocytic NADPH oxidase activation in response to microbial infection or TLR stimulation. Cell Host Microbe 11: 264-276.

7. Yang CS, Yuk JM, Lee YH, Jo EK (2016) Toxoplasma gondii GRA7-Induced TRAF6 Activation Contributes to Host Protective Immunity. Infect Immun 84: 339-350.

8. Song Y, Kim YR, Kim SM, Ul Ain Q, Jang K, et al. (2016) RNAi-mediated silencing of TNF-alpha converting enzyme to down-regulate soluble TNF-alpha production for treatment of acute and chronic colitis. J Control Release 239: 231-241.
